# Simultaneous Multiple Resonance Frequency imaging (SMURF): Fat‐water imaging using multi‐band principles

**DOI:** 10.1002/mrm.28519

**Published:** 2020-09-27

**Authors:** Beata Bachrata, Bernhard Strasser, Wolfgang Bogner, Albrecht Ingo Schmid, Radim Korinek, Martin Krššák, Siegfried Trattnig, Simon Daniel Robinson

**Affiliations:** ^1^ High Field MR Centre, Department of Biomedical Imaging and Image‐Guided Therapy Medical University of Vienna Vienna Austria; ^2^ Christian Doppler Laboratory for Clinical Molecular MR Imaging Vienna Austria; ^3^ Athinoula A. Martinos Center for Biomedical Imaging, Department of Radiology Massachusetts General Hospital, Harvard Medical School Boston MA USA; ^4^ High Field MR Centre, Center for Medical Physics and Biomedical Engineering Medical University of Vienna Vienna Austria; ^5^ Institute of Scientific Instruments of the CAS Brno Czech Republic; ^6^ Department of Internal Medicine III, Division of Endocrinology and Metabolism Medical University of Vienna Vienna Austria; ^7^ Centre of Advanced Imaging University of Queensland Brisbane QLD Australia; ^8^ Department of Neurology Medical University of Graz Graz Austria

**Keywords:** chemical shift artefact, dual‐band, fat‐suppression, fat‐water imaging, Simultaneous Multiple Resonance Frequency (SMURF), spatial‐spectral pulses

## Abstract

**Purpose:**

To develop a fat‐water imaging method that allows reliable separation of the two tissues, uses established robust reconstruction methods, and requires only one single‐echo acquisition.

**Theory and Methods:**

The proposed method uses spectrally selective dual‐band excitation in combination with CAIPIRINHA to generate separate images of fat and water simultaneously. Spatially selective excitation without cross‐contamination is made possible by the use of spatial‐spectral pulses. Fat and water images can either be visualized separately, or the fat images can be corrected for chemical shift displacement and, in gradient echo imaging, for chemical shift‐related phase discrepancy, and recombined with water images, generating fat‐water images free of chemical shift effects. Gradient echo and turbo spin echo sequences were developed based on this Simultaneous Multiple Resonance Frequency imaging (SMURF) approach and their performance was assessed at 3Tesla in imaging of the knee, breasts, and abdomen.

**Results:**

The proposed method generated well‐separated fat and water images with minimal unaliasing artefacts or cross‐excitation, evidenced by the near absence of water signal attributed to the fat image and vice versa. The separation achieved was similar to or better than that using separate acquisitions with water‐ and fat‐saturation or Dixon methods. The recombined fat‐water images provided similar image contrast to conventional images, but the chemical shift effects were eliminated.

**Conclusion:**

Simultaneous Multiple Resonance Frequency imaging is a robust fat‐water imaging technique that offers a solution to imaging of body regions with significant amounts of fat.

## 1 INTRODUCTION

The human body contains essential fat, which is distributed over the whole body and is crucial for its function, and subcutaneous and visceral fat, which serve as energy reserves. The fat signal comprises many spectral components, but because about 80% of the total signal stems from peaks with shifts between 3.2 and 3.9 ppm from water,^1^ it is often considered, for practical purposes, as having only a single peak with a 3.5 ppm shift relative to water.^2^ Visualization of fatty tissues provides valuable insights into physiology and pathology, but the chemical shift of fat with respect to water gives rise to artifacts. First, it leads to a displacement of the fat image relative to the water image along all frequency‐encoding directions (Type 1 chemical shift artifact). Second, in gradient echo (GRE) imaging, interference between the fat and water signals, which are subject to different phase evolutions, leads to complex signal cancellation when acquired at any time other than in‐phase echo times (TEs) (Type 2 chemical shift artifact). Moreover, the shorter T_1_ and longer T_2_ relaxation times of fat lead it to appear hyperintense, obscuring the water signal of primary interest.

In some cases, it may be sufficient to suppress the fat signal. Fat‐suppression techniques either take advantage of the differences between the relaxation times of fat and water, such as the short inversion time inversion recovery (STIR) method,^3^ or of their chemical shift difference, such as spectrally selective water excitation^4^ and spectrally selective fat‐saturation^5^ methods. In other contexts, however, the fat must be visualized in order to be able to assess the anatomy or pathology of interest. Cartilage thickness, for instance, is optimally determined with respect to the bone, in which most of the signal derives from the fatty marrow. The soft tissue lesions embedded in fatty tissues are likewise best assessed in combined fat‐water images,^6^ but chemical shift effects make such an assessment difficult.

The Dixon method^7^ is the only approach in common use that simultaneously generates separate images of fat and water. It takes advantage of the Type 2 chemical shift artifact; the fact that fat and water signals add in a complex fashion, with the result being dependent on the echo time. In its simplest embodiment, two echoes are acquired at TEs at which fat and water signals are in‐phase and out‐of‐phase. Separate images of fat and water are generated by their summation and subtraction, respectively. The Dixon approach, like other methods based on the chemical shift, is sensitive to inhomogeneity in the static magnetic field: the phase errors caused by ΔB_0_ lead to incomplete fat‐water separation unless an effective correction is applied. In the past decades, the Dixon approach has been improved by the use of a larger number of echoes with different fat‐water phase relationships.^8^ Unlike the original two‐point Dixon, three‐point Dixon^9^ allows the identification and correction of phase errors smaller than 2π, which corresponds to the requirement that ΔB_0_ be less than half of the fat‐water chemical shift difference. Phase unwrapping or an analytical method can be used for larger phase discrepancies, but phase unwrapping^10^, ^11^, ^12^ is generally time‐consuming and prone to errors, and analytical approaches^13^, ^14^, ^15^, ^16^ require at least three echoes and additional processing such as the use of region‐growing algorithms.^15^ To reduce acquisition times, which are generally relatively long due to the need to acquire multiple echoes, high receiver bandwidths (rBWs) are usually used, despite the deleterious effect this has on image signal‐to‐noise ratio (SNR) and restrictions it poses on the minimum field‐of‐view (FOV). Acquisitions with bipolar readout can be used alternatively (or additionally) although this necessitates error‐prone corrections for eddy currents.^17^, ^18^ Dixon approaches are sensitive to flow‐related phase artifacts^8^, ^19^ and rely on the assumptions that there is either no $T_{2}^{*}$ decay or identical $T_{2}^{*}$ decay for fat and water signals. Although the Dixon method has been continuously improved since its introduction 35 years ago, fat‐water swaps still frequently occur^20^, ^21^, ^22^ and may lead to image misinterpretation.

The aim of this study was to develop an alternative, more reliable method of simultaneously but separately imaging fat and water that would allow the generation of images free of chemical shift artifacts.

## 2 THEORY

**FIGURE 1:**
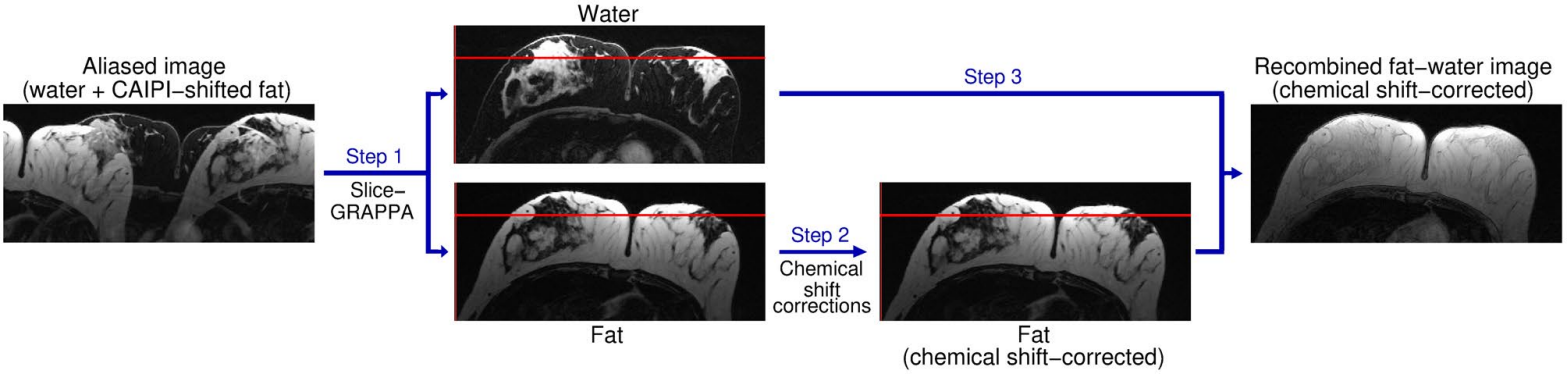
Fat‐water separation, chemical shift corrections, and recombination in Simultaneous Multiple Resonance Frequency imaging. Overlapping water and CAIPIRINHA‐shifted fat images are unaliased using slice‐GRAPPA (Step 1). The fat image is shifted to reverse chemical shift displacement—see the positions of fat before and after the displacement correction relative to the red reference line—and, for GRE acquisitions, corrected for chemical shift‐related phase evolution (Step 2). The fat and water images are then recombined (Step 3), either in complex or magnitude‐only, generating a fat‐water image free of chemical shift effects.

In the proposed method, dual‐band pulses^23^, ^24^ are used to simultaneously excite two species with different resonance frequencies, such as fat and water. In 2D and slab‐selective 3D imaging, spatial‐spectral pulses^25^ are employed to achieve both spectral and spatial selectivity. Images of one of the species are shifted along the phase‐encoding direction(s) using CAIPIRINHA,^26^ allowing them to be separated using parallel imaging techniques.^27^, ^28^ Separate fat and water images can either be considered individually or, after correction of chemical shift displacement and phase discrepancy, recombined (Figure 1). Because of the similarities to Simultaneous Multi‐Slice (SMS) imaging, we refer to this approach as Simultaneous Multiple Resonance Frequency (SMURF) imaging.^29^, ^30^

### 2.1 SMURF excitation

In 2D and slab‐selective 3D acquisitions, in which a gradient is applied during the radiofrequency (RF) excitation to achieve slice/slab selection, spatial‐spectral pulses^25^ are required for SMURF imaging. Using the small tip‐angle approximation and the concept of excitation k‐space outlined by Pauly,^31^ the transversal magnetization *M_xy_* excited by a spatial‐spectral pulse *B_SS_(t)* of duration *T* is defined as

(1) $$M_{\mathrm{xy}}\left(z,\;f\right)=i\gamma M_{0}\int_{0}^{T}B_{\mathrm{SS}}(t)e^{tk_{z}(t)z‐ik_{f}(t)f}dt,$$

where *M*_0_ is the initial magnetization,

(2) $$k_{z}\left(t\right)=\gamma \int_{T_{z}}^{t}G_{z}\left(s\right)ds,$$

where *G_z_* is the slice‐selective gradient, and

(3) $$k_{f}\left(t\right)=T‐t.$$

**FIGURE 2:**
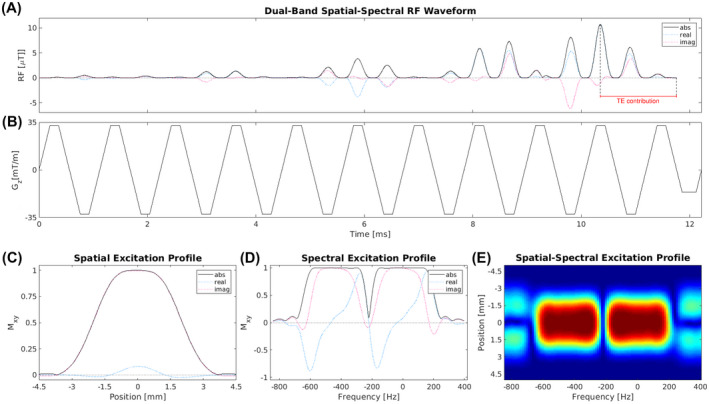
Dual‐band spatial‐spectral pulse used for 90° excitation in 2D TSE‐SMURF imaging. A, RF waveform created by a train of sinc subpulses modulated by a dual‐band envelope. B, Waveform of the oscillating trapezoidal z‐gradient played concurrently with the RF pulse for slice‐selection. Excitation profile of the RF pulse, comprising two frequency bands offset by 440 Hz, shown as a function of the position along the slice‐selection direction (C), the Larmor frequency (D), and the Larmor frequency and position along the slice‐selection direction (E).

Spatial‐spectral pulses comprise a train of short subpulses modulated by a temporal weighting function (Figure 2A). The concurrently applied *G_z_* typically consists of a series of short trapezoids of alternating polarity (Figure 2B), the duration of which (*T_z_*) determines the separation between the excited and suppressed frequencies (*f_s_*) (Figure 2E), as well as the frequencies of inherent periodic replicates.^25^ The spatial excitation profile is thus determined by the Fourier transform of the subpulses and the spectral excitation profile is determined by the Fourier transform of the temporal weighting function. To use the acquisition time and applied gradients most efficiently, the subpulses can be applied during both positive and negative phases of the *G_z_* (true null design), and also during gradient ramping. The duration of the gradient sublobes has to be chosen such that the frequencies of periodic replicates and the suppressed frequencies, for the true null design given by

(4) $$f_{s}=\frac{1}{4T_{z}},$$

do not impair the desired spectral excitation profile. To allow the longest *T_z_* (maximal *G_z_* area) and hence, the minimum slice thickness, *f_s_*, should be equal to the chemical shift difference. In SMURF fat‐water imaging at 3 T, this requires a sublobe duration of about 0.56 ms.

To achieve simultaneous but separate spatially selective imaging of two chemical species with different Larmor frequencies, a dual‐band temporal weighting function is applied to the spatial‐spectral pulses. This is created by superimposing two single‐band functions of waveforms *b_SB_* (in our case identical) and amplitude weighting factors *A*:

(5) $$B_{\mathrm{DB}}=A_{1}b_{\mathrm{SB}}+A_{2}b_{\mathrm{SB}}e^{i\Delta ft},$$

with the frequency offset between the bands (Δf) given by the chemical shift difference. The excited magnetization profile of such a dual‐band spatial‐spectral pulse is given by

(6) $$M_{\mathrm{xy}}\left(z,\;f_{1},\;f_{2}\right)=i\gamma M_{0}\int_{0}^{T}b_{\mathrm{SB}}(t)e^{tk_{z}(t)z}\left(A_{1}e^{‐ik_{f}(t)f_{1}}+A_{2}e^{‐ik_{f}(t)(f_{1}+\Delta f)}\right)dt.$$

Since the flip angle (α) of an RF pulse is defined as

(7) $$\alpha (t)=\gamma \int_{0}^{T}Ab_{\mathrm{SB}}(t)dt,$$

Equation (6) shows that the separate excitation in SMURF imaging offers the possibility of exciting both chemical species at their respective Ernst angles. This can lead to an improvement of SNR compared to a broadband excitation if their T_1_ values are significantly different, as is the case for fat (which has a T_1_ of 400 ms at 3 T^32^) and muscle (which is mainly composed of water and has a T_1_ of 1400 ms at 3 T^32^).

The single‐band function needs to have sufficient spectral selectivity to avoid cross‐excitation between the two species, but a wide and homogeneous spectral passband to provide some insensitivity toward field inhomogeneity. Such selective, analytically defined excitation profiles can be designed using the Shinnar‐Le Roux (SLR) algorithm,^33^, ^34^ which allows a trade‐off between the pulse duration (*T*), bandwidth (*B_W_*), transition width (*B_w_W*), and the passband ($\delta_{p}$) and stopband ($\delta_{s}$) ripples,^35^ following

(8) $$D_{\infty }\left(\delta_{p},\delta_{s}\right)=TB_{w}W.$$

The narrow transition width required for SMURF fat‐water imaging restricts the minimum duration and/or ripples of the temporal weighting function. To achieve high spectral selectivity at reasonable echo times and echo spacings, minimum‐phase SLR pulses can be used. These are shorter than linear‐phase pulses of the same spectral selectivity and, additionally, their contribution to the TE (ie, isodelay) is much less than half of the pulse width (Figure 2A).

In a spatially non‐selective 3D acquisition, no gradients are applied during the excitation, reducing Equation (6) to

(9) $$M_{\mathrm{xy}}\left(f_{1},\;f_{2}\right)=i\gamma M_{0}\int_{0}^{T}B_{\mathrm{SB}}(t)\left(A_{1}e^{‐ik_{f}\left(t\right)f_{1}}+A_{2}e^{‐ik_{f}\left(t\right)(f_{1}+\Delta f)}\right)dt.$$

The spectrally selective dual‐band pulses can, therefore, be used directly for SMURF imaging.

### 2.2 Controlled aliasing using CAIPIRINHA

Although they are excited separately, fat and water signals that originate from the same position will generally overlap in the image. The CAIPIRINHA technique^26^ can be used to shift one of the images along the phase‐encoding direction(s). To shift the image by the desired fraction of the FOV $\left(\Delta y\right)$, the phase between adjacent k‐space lines has to increase linearly by

(10) $$\varphi =\frac{2\pi \Delta y}{\mathrm{FOV}}.$$

Overlapping signals are thereby modulated by different coil sensitivities, allowing them to be separated using parallel imaging techniques.^27^ As in SMS, the parallel imaging reconstruction decreases image SNR by the g‐factor,^36^ which reflects the geometry of the receiver coil array and the applied CAIPIRINHA shift. A shift of half of the FOV between the two images is generally advantageous, as it leads, for many coils, to a high coil sensitivity difference between the overlapping voxels and thereby low g‐factor penalty. To CAIPIRINHA‐shift the fat by FOV/2, the phase of the fat band of the dual‐band pulse needs to be incremented by π for each successive k‐space line, which can be achieved with phase angles of (0, π, 2π, 3π, …), which is equivalent to (0, π, 0, π, …) because of phase wrapping. If parallel‐imaging acceleration is also used, a FOV/3 shift is generally preferable for even acceleration factors, despite a higher g‐factor SNR penalty, because it avoids complete overlap between undersampling artifacts and the CAIPIRINHA‐shifted image. This can be achieved using 2/3π phase jumps between k‐space lines, that is, (0, 2π/3, 4π/3, 0, …). In TSE imaging, the CPMG condition,^37^, ^38^ which requires that there be a π/2 phase angle between the excitation and refocusing pulses, poses further requirements on SMURF pulses. Although both broadband and spectrally selective refocusing pulses can generally be used in TSE‐SMURF imaging, if a FOV/3 shift is desired, the CPMG condition can only be satisfied for both fat and water by using spectrally selective refocusing pulses; the phase of the individual pulse bands can be modified to follow the phase pattern of the excitation, although this increases the minimum echo spacing.

### 2.3 Chemical shift corrections

Once separated, the fat signal can be corrected for the chemical shift displacement of N_x_ voxels (Type 1 chemical shift artifact correction), where

(11) $$N_{x}=\frac{\Delta f}{rBW/pixel},$$

and, for GRE acquisitions, for the phase discrepancy (Type 2 chemical shift artifact correction) of

(12) $$\Delta \phi \left(\mathrm{TE}\right)=\;\text{mod}\;\left(TE,\left(\frac{1}{\Delta f}\right)\right)2\pi \Delta f,$$

where Δf is the chemical shift difference and *rBW/pixel* the receiver bandwidth per pixel, before a complex summation, that is, recombination, with the water signal (Figure 1). This generates a fat‐water image as obtained using broadband acquisition at an in‐phase TE and infinitely high rBW. The complex recombination allows the concurrent generation of an opposite‐phase image that can be used for improved image segmentation or visualization of water‐lesions embedded in fatty tissues and vice versa. Assuming Gaussian noise distribution of the signal with a zero mean,^39^ the complex addition of two images is expected to cause noise enhancement by a factor of $\sqrt{2}$, that is, ~1.4.^40^ Alternatively, the magnitudes images can be added. Magnitude summation removes the need for Type 2 chemical shift artifact correction and eliminates any residual fat‐water signal cancellation that might result from phase not being fully refocussed at the echo time (due to $\Delta B_{0}$ or eddy currents, for instance), thus yielding images that are more fully in‐phase than those from a conventional (broadband) acquisition at in‐phase echo time. The magnitude summation, however, results in noise enhancement by a factor of 2.

## 3 METHODS

Gradient‐recalled echo (GRE) and turbo spin echo (TSE) fat‐water SMURF (Simultaneous Multiple Resonance Frequency) sequences were developed. All measurements were performed with a 3T Siemens Prisma scanner (*syngo* MR VE11C, Siemens Healthineers, Erlangen, Germany). A phantom consisting of a cylinder filled with cream with a 36% fat content placed inside a larger, water‐filled cylinder was scanned using a 20‐channel head coil (Siemens Healthineers) to evaluate the effectiveness of chemical shift artifact corrections and quantify SNR. Eleven healthy volunteers were scanned to assess the performance of the proposed approach in a number of body regions and to compare SNR and SNR efficiency ($SNR/\sqrt{\text{scan time}}$)^41^ with the Dixon method in vivo. Comparison between SMURF and several of the most often used, two‐point and three‐point Dixon methods was performed. First, 3D two‐point GRE Dixon images were acquired using the Siemens product RF‐spoiled GRE VIBE^42^ sequence. In the absence of a vendor three‐point GRE Dixon sequence for *syngo* MR VE11C, 2D three‐point GRE images were acquired using a standard (non‐Dixon) Siemens product GRE sequence and reconstructed offline. Finally, for TSE, two‐point Dixon images were acquired using the Siemens product sequence (“tse_dixon”). The left knee of four volunteers (V1‐V3, V11) was scanned using a 15‐channel knee coil (QED, Mayfield Village, OH, USA). In the absence of a dedicated breast coil, both breasts of three volunteers (V4‐V6) and the abdominal region of four volunteers (V7‐V9, V10) were scanned using an 18‐channel flex‐coil (Siemens Healthineers) in combination with a spine coil array (Siemens Healthineers). Written informed consent was provided by all the participants, and the study was approved by the Ethics Committee of the Medical University of Vienna.

### 3.1 RF pulse design

Two 11.76 ms least‐squares filtered minimum‐phase Shinnar‐Le Roux pulses^33^, ^34^, ^35^, one optimized for excitation at 90° and used in TSE imaging and the second for excitation at 30° and used in GRE imaging, were designed using Vespa^43^, ^44^ and used to create both bands of the corresponding dual‐band pulse, with a frequency offset of 440 Hz. The resulting dual‐band pulse was used for excitation for non‐selective 3D acquisitions. For 2D acquisitions, the spatial‐spectral pulse (Figure 2 and Supporting Information Figure S1, which is available online) created by a train of sinc subpulses with a time‐bandwidth product of 2.3, modulated by the dual‐band envelope described above, was applied concurrently with a trapezoidal oscillating slice‐selective gradient with a period of 1.12 ms (as described in the Theory section) and a ramp time of 0.20 ms to comply with the system limits on maximal slew rates (180 T/m/s) for the maximum *G_z_* (34 mT/m) allowed. The subpulses were applied during both positive and negative gradient sublobes and gradient ramping, allowing a minimum slice thickness of 3.0 mm. For TSE, a broadband refocusing pulse from a Siemens pulse library (SE2560A180.SE180_12A2_2) was used. For all phantom measurements, 16.24 ms linear phase SLR (firstly designed and used during the process of SMURF development) was used instead, resulting in increased minimum TEs.

### 3.2 Acquisition parameters

#### 3.2.1 Phantom measurements

##### Effectiveness of chemical shift corrections

Fifty one measurements were made with the 2D GRE SMURF sequence, with TEs ranging from 12.5 to 17.5 ms in 0.1 ms increments. Five coronal slices were measured with left‐right phase‐encoding direction, FOV = 240 × 240 mm, resolution = 1.0 × 1.0 × 3.5 mm, 20% slice gap, repetition time (TR) = 200 ms, FA = 43° and rBW/pixel = 440 Hz.

##### SNR comparison between SMURF and conventional imaging

A total of 75 scans were acquired; 25 repetitions of scans using each of three variants of 2D GRE acquisitions: (a) SMURF with CAIPIRINHA‐shifted fat, (b) SMURF without CAIPIRINHA‐shifting, and (c) “conventional” acquisitions (using broadband excitation and no spectral saturation). For each scan, five coronal slices were acquired with left‐right phase‐encoding direction, FOV = 144 × 144 mm, resolution = 0.75 × 0.75 × 3.5 mm, 50% slice gap, TE = 13.8 ms, TR = 200 ms, FA = 41° and rBW/pixel = 150 Hz.

For both phantom protocols, conventional low‐resolution 2D GRE scans were acquired as sensitivity reference scans for parallel imaging reconstruction of separate fat and water images.

#### 3.2.2 In vivo measurements

##### Separation/suppression and image quality

To compare SMURF with state‐of‐the‐art fat‐water suppression and separation techniques, water‐saturated (WaterSat), fat‐saturated (FatSat), and Dixon images were also acquired. Water‐saturated and fat‐saturated acquisitions used pure CHESS suppression with a Gaussian presaturation pulse of BW = 375 Hz, *t* = 5.12 ms, FA depending on TR,^45^ and with a frequency offset between the imaged and suppressed frequency bands of 407 Hz. Conventional low‐resolution multi‐echo (TE = {2.3, 4.6} ms) GRE scans were acquired to assess the B_0_ inhomogeneity and the first echo images were used as sensitivity reference scans for fat‐water separation using parallel imaging reconstruction. To evaluate possible changes to image contrast and quality resulting from the use of SMURF, high‐resolution conventional fat‐water images were also acquired. For all in vivo measurements, two to three iterations of image‐based second‐order B_0_ shimming procedure were carried out.

###### Knee

Sagittal 2D TSE images were acquired with anterior‐posterior phase‐encoding direction, FOV = 160 × 160 mm, resolution = 0.5 × 0.5 × 3 mm, 36 slices, 10% slice gap, echo train length of ETL = 4, TR = 2500 ms, refocusing FA = 150°, rBW/pixel = 150 Hz, and monopolar readout. SMURF images were acquired with an echo spacing of 11.94 ms and TA = 3:17 min. For comparison, two‐point Dixon images were acquired with an echo spacing of 14 ms (the minimum possible) and TA = 6:35 min. Additionally, three images using broadband excitation were acquired: (a) conventional (no spectral saturation), (b) WaterSat, (c) FatSat, all with an echo spacing of 12 ms and TA = 3:17 min. Finally, GRE SMURF and GRE Dixon images of a knee of one volunteer (V11) were acquired (see Supporting Information Figure S6).

###### Breasts

Volunteers were positioned prone, padded with pillows so that the breasts were close to the coils. Transversal 2D GRE images were acquired in single breathholds (to avoid motion artifacts due to the non‐standard measurement setup) and with left‐right phase‐encoding direction, matrix size = 320 × 320, an in‐plane resolution between 1.0 × 1.0 and 1.05 × 1.05 mm (varying between the subjects), 3 mm slice thickness, all using phase partial Fourier factor of 6/8 and monopolar readout. The SMURF images of six slices with 20% slice gap were acquired with TE = 6.8 ms, TR = 110 ms, TA = 26.3 s, rBW/pixel = 240 Hz, using respective Ernst angles for fat and water bands, FA(fat) = 42° and FA(water) = 22°. For comparison, two different Dixon images were acquired: (a) slab‐selective 3D dual‐echo GRE Dixon images (3D two‐point Dixon ‐ VIBE^42^) with eight slices (the minimum number possible), TE = {2.27, 5.67} ms, TR = 8 ms, TA = 23.0 s, FA = 9°, rBW/pixel = 490 Hz; and (b) 2D triple‐echo GRE Dixon images (2D three‐point Dixon) images with six slices (the maximum number possible in one breathhold) with 20% slice gap, TE = {2.2, 5.3, 8.4} ms, TR = 110 ms, TA = 26.3 s, FA = 32°, rBW/pixel = 540 Hz for offline reconstruction. Additionally, three images using broadband excitation were acquired: (a) “conventional” (no spectral saturation) with six slices, FA = 32°; (b) WaterSat, four slices (the maximum number possible within one breathhold), FA(fat) = 42°; (c) FatSat, four slices, FA(water) = 22°; all with 20% slice gap, TE = 6.8 ms, TR = 110 ms, TA = 26.3 s, rBW/pixel = 240 Hz as for SMURF. The flip angles (FAs) used in the Dixon and conventional acquisitions represented a mean of the given fat and water Ernst angles. Shimming was performed over the full extent of the breasts.

###### Abdomen

Transversal 2D GRE SMURF images were acquired in a single breathhold with anterior‐posterior phase‐encoding direction, using the same protocol as for breast measurements other than the FOV, which led to an in‐plane resolution between 1.1 × 1.1 mm and 1.2 × 1.2 mm (varying between the subjects) and to slightly higher water FA, FA(water) = 24°, in the light of different T_1_.

##### SNR and SNR efficiency comparison between SMURF and Dixon imaging

Four pairs of identical 2D GRE SMURF and four pairs of each of two variants of identical 2D three‐point GRE Dixon abdominal images of one volunteer (V10) were acquired, interleaving the pairs of SMURF with the pairs of Dixon images. SMURF and one set of Dixon images (“long‐TR”) were acquired with the same parameters as the data for comparison of in vivo separation/suppression quality and the second set (“short‐TR”) with the lowest possible TR = 67 ms, TA = 16.0 s, and FA = 26° (mean of fat and water Ernst angles).

### 3.3 Data analysis

SMURF fat and water images were reconstructed from raw data in MATLAB (Mathworks Inc, Natick, MA) using slice‐GRAPPA^24^ with the conventional low‐resolution scans to calculate the GRAPPA kernel.

#### 3.3.1 Phantom measurements

##### Effectiveness of chemical shift corrections

The phase difference relative to water at the TE (Equation 12) was added to the fat signal, which was shifted to reverse the chemical shift displacement (Equation 11) (correction performed in k‐space). An ROI within the cream was defined and the time courses of the mean signal over the ROI in the not‐corrected and phase‐corrected (ie, Type 2 chemical shift artifact‐corrected) recombined fat‐water images were compared.

##### SNR comparison between SMURF and conventional imaging

Two regions of interest (ROIs) were defined manually using MRIcro^46^; one within the cream (“cream ROI”) and the second within the water area of the phantom in which there was no CAIPIRINHA‐shifted fat signal (“water ROI”). The SMURF images with CAIPIRINHA‐shifting were unaliased and recombined without chemical shift corrections. For each sequence variant, the SNR of fat‐water images was calculated over the 25 acquisitions on a pixel‐by‐pixel basis,^47^, ^48^ and mean values over the ROIs were calculated. Additionally, to assess the influence of individual SMURF reconstruction steps, the mean SNR was calculated over the water ROI in (a) aliased images with CAIPIRINHA‐shifted fat, (b) unaliased water images, and (c) recombined fat‐water images.

#### 3.3.2 In vivo measurements

##### Separation/suppression and image quality

To assess if the field homogeneity met the SMURF requirement to selectively excite fat and water, maps of ΔB_0_ (fieldmaps)^49^ were calculated from the low‐resolution GRE scans. The effectiveness of fat‐water separation with SMURF imaging was evaluated visually and compared with water‐saturated images, fat‐saturated images, and Dixon results. The recombined SMURF fat‐water images were compared with the conventional (broadband excitation) images to evaluate the effect of the chemical shift displacement correction, possible changes to image contrast, and other aspects of image quality. No Type 2 chemical shift artifact correction was applied in this case as in‐phase acquisitions were used to achieve the desired contrast, both for SMURF and conventional images. Note that the images from different acquisitions (ie, SMURF, conventional, WaterSat, FatSat, Dixon) were not coregistered, in order to avoid introducing blurring and therefore, particularly in breathhold acquisitions, there are minor discrepancies between slice positions. The 3D two‐point GRE Dixon images were calculated from the two acquired echoes using the online reconstruction of the VIBE sequence and the 2D three‐point Dixon images were calculated offline, using the graph‐cut approach^50^ from the Fat‐water Toolbox.^51^ In the knee, the 2D two‐point TSE Dixon images were calculated using the online reconstruction of the sequence.

##### SNR and SNR efficiency comparison between SMURF and Dixon imaging

The SNR and SNR efficiency of SMURF and Dixon were assessed in the separated fat and water images over 40 manually defined ROIs of 100 voxel size^52^; 20 in the water images, within the water‐dominated tissue areas (10 in erector spinae muscles, 5 in the liver and 5 in kidneys), and 20 in the fat images, within fat‐dominant tissue areas (10 in subcutaneous and 10 in visceral adipose tissue). The Dixon images were calculated offline from the multi‐echo GRE (three‐point Dixon) images using the graph‐cut approach^50^ from the Fat‐water Toolbox.^51^ No coregistration between the separate breathhold acquisitions was applied; the ROIs were defined in the areas with the least motion between the individual acquisitions and no fat‐water swaps. The SNR was calculated as proposed in Ref 48; the average and difference images from pairs of repeated acquisitions were calculated and the “signal” was calculated as the mean pixel value over the ROI in the average image and the “noise” was calculated as the standard deviation over the same ROI in the difference image divided by $\sqrt{2}$ to correct for the increased variance due to image subtraction.^52^ The median values over the four repetitions and over all of the ROIs localized in the same tissues were calculated and compared between the methods.

## 4 RESULTS

**FIGURE 3:**
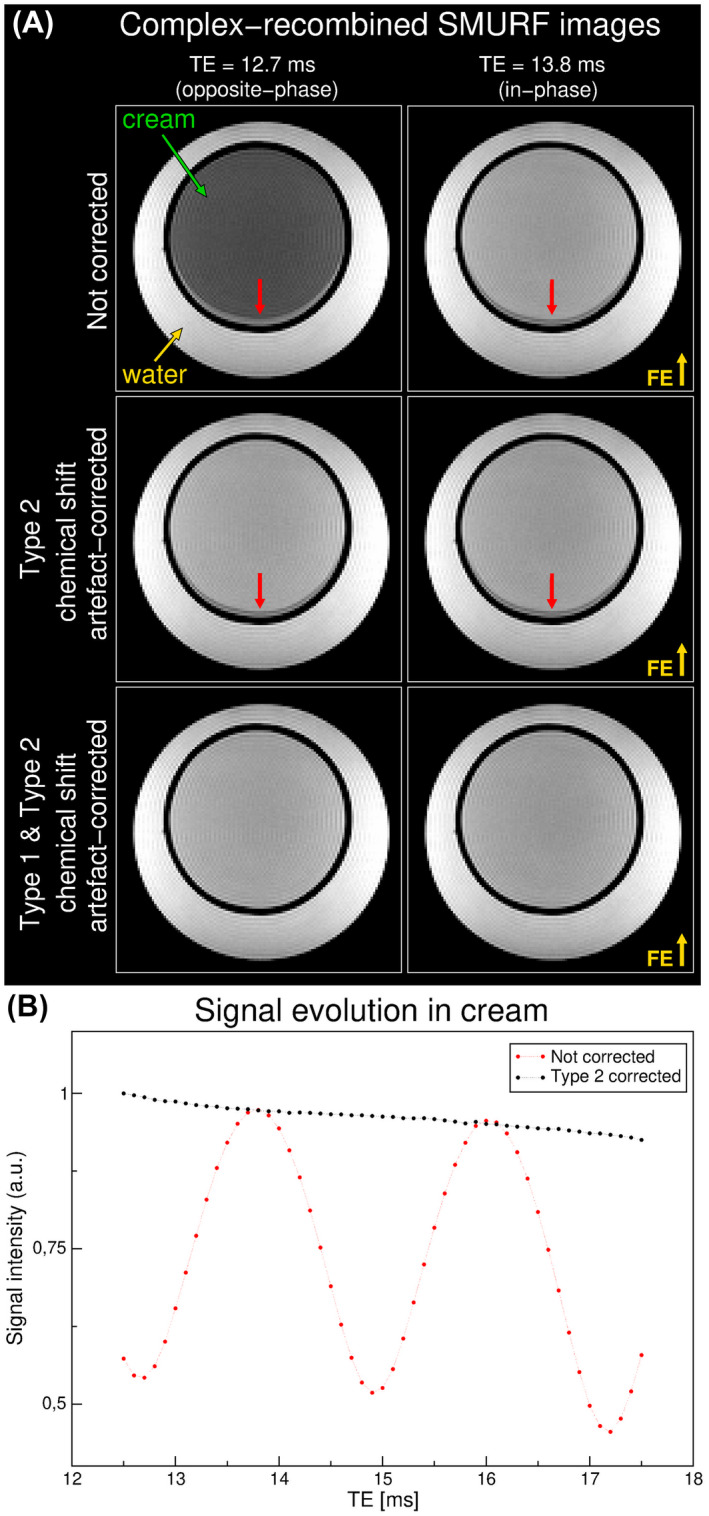
The correction of Type 1 and Type 2 chemical shift artifacts in a cream‐water phantom. A, The not‐corrected images (upper row) show reduced signal at the out‐of‐phase TE (TE = 12.7 ms; top left) compared to the in‐phase TE (TE = 13.8 ms, top right) and the presence of chemical shift displacement artifact (red arrows) in the frequency‐encoding (FE) direction. The phase correction for Type 2 chemical shift artifact removes the signal dephasing at the out‐of‐phase TE (middle row). The misalignment between fat and water is removed by Type 1 chemical shift artifact correction (bottom row). B, The mean signal intensity over an ROI in the cream shows oscillating behavior over TE (red dots), which is removed by the phase correction for the Type 2 chemical shift artifact (black dots), in addition to exponential signal decay.

In the cream‐water phantom, an oscillating intensity of the cream signal over TE, with a period of roughly 2.3 ms, was observed in the complex recombined GRE SMURF images without phase correction for Type 2 chemical shift artifact (Figure 3B). The correction removed the oscillatory behavior. The Type 1 chemical shift artifact correction removed the misalignment between fat and water (Figure 3A, third row).

The GRE images of the cream‐water phantom acquired with SMURF excitation but no CAIPIRINHA‐shifting (hence no parallel imaging reconstruction) had similar SNR to the conventional images (broadband excitation with no spectral saturation) (Table 1A). The recombined SMURF images, reconstructed from the aliased water and CAIPIRINHA‐shifted fat images, showed a decrease in SNR by a factor of 1.7 ‐ 2.0 (varying between ROIs) compared to the conventional images. This comprised two effects:  parallel imaging reconstruction which, in the water ROI, resulted in an SNR decrease by a factor of 1.2; and  the recombination of the separated images, which led to a further SNR decrease by a factor of 1.4 (Table 1B). The signal intensities were similar in all images; SNR differences were attributable to changes in noise.

**TABLE 1:** (A) Mean SNR values over the water and cream ROIs in (a) conventional (broadband excitation) GRE images, (b) GRE SMURF images without CAIPIRINHA‐shifting, and (c) recombined GRE SMURF fat‐water images of the cream‐water phantom. The SNR of conventional and no‐CAIPI SMURF images was similar, whereas that of recombined SMURF images was markedly lower. (B) Mean SNR values over the water ROI (in which there was no fat present in all cases) in (a) aliased SMURF fat‐water images, (b) unaliased SMURF water images and (c) recombined SMURF fat‐water images of the cream‐water phantom. SNR in aliased SMURF and conventional images was similar (A), top row), but was reduced in unaliased water images and even further reduced in recombined images. The same TR and TA were used for all the measurements, resulting in the same relative decreases in SNR efficiency between the methods.

SNR in cream‐water phantom				
	Water	Cream		Water
Conventional	97.7	74.9	Aliased SMURF	97.8
No‐CAIPI SMURF	97.2	76.2	Unaliased SMURF water	82.2
Recombined SMURF	58.6	38.2	Recombined SMURF	58.6
	(A)		(B)	

**FIGURE 4:**
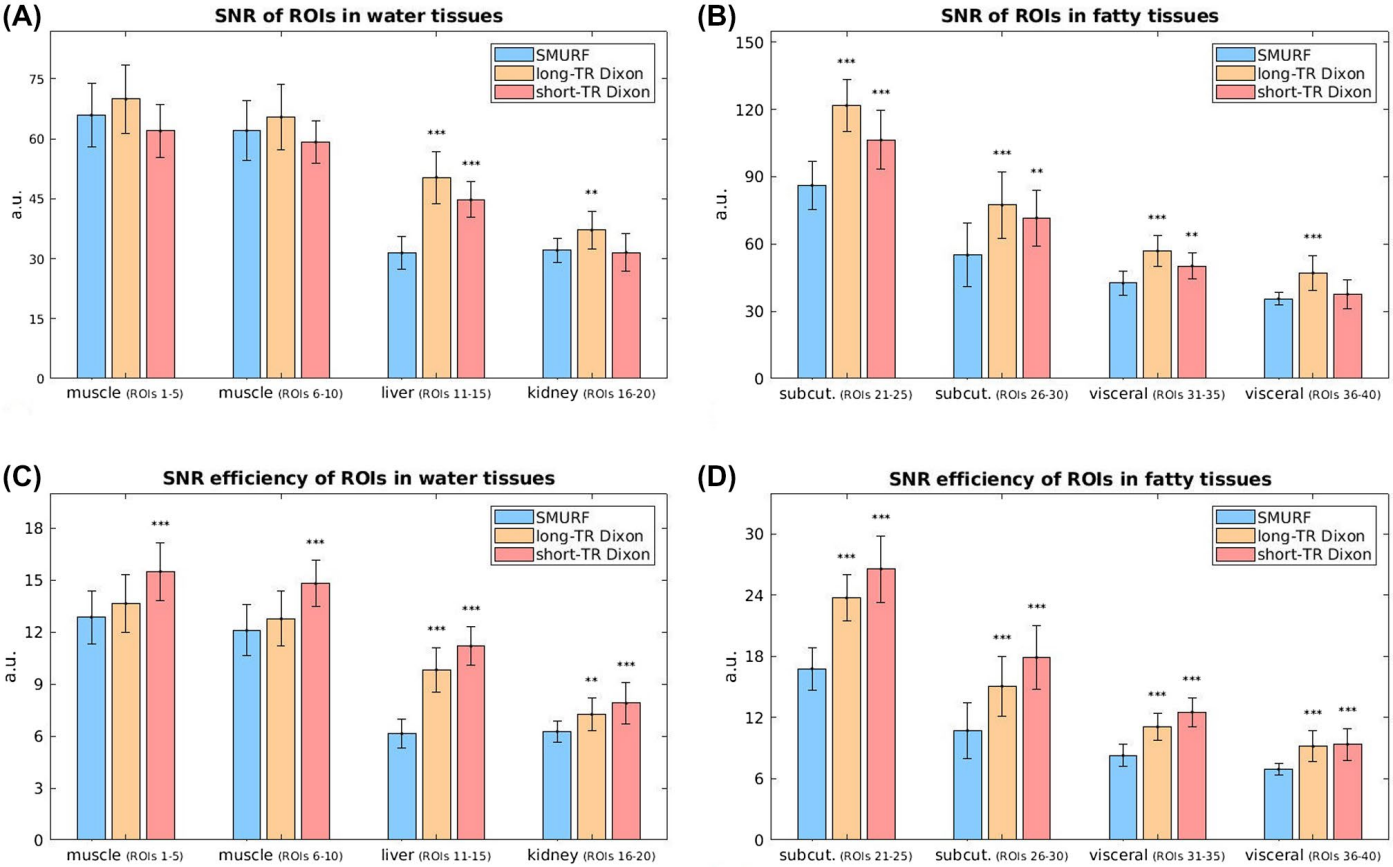
Comparison of SNR (upper row) and SNR efficiency (bottom row) between SMURF (A), long‐TR Dixon (B), and short‐TR Dixon (C), assessed in separate water and fat images. Bar lengths correspond to median values over all ROIs localized in the same area and over the four repetitions. Error bars represent the median absolute deviation of the individual values. Upper row) SNR was slightly lower in SMURF than in the long‐TR Dixon (by a factor of about 1.1 in water (except liver) (A) and 1.4 in fat (B)). (Bottom row) The SNR efficiency of SMURF was generally lower than that of the Dixon approach; by a factor of about 1.4 and 1.6 compared to the short‐TR Dixon for water (except liver) (C) and fat (D), respectively. The largest difference in SNR and SNR efficiency between SMURF and the Dixon approach was observed in the liver (ROIs 11‐15); by a factor of about 1.7 compared to the short‐TR Dixon. (The “*” depict statistically significant differences between SMURF and the respective Dixon approach (“*” for *P* < .05, “**” for *P* < .01 and “***” for *P* < .001).)

In vivo, the SNR of GRE SMURF was slightly lower than that of the long‐TR three‐point GRE Dixon (which had the same TR as SMURF); medians decreased by a factor of about 1.1 in water (except liver) and 1.4 in fat. The SNR efficiency of SMURF was lower than that of the short‐TR three‐point Dixon (the more SNR efficient of the two variants), by factors of 1.4 and 1.6 for water (except liver) and fat, respectively. The largest difference in SNR and SNR efficiency between SMURF and the Dixon approach was observed in the liver, in which it was decreased by a factor of about 1.7 compared to the short‐TR Dixon (Figure 4, Supporting Information Figure S2 with Table S1).

**FIGURE 5:**
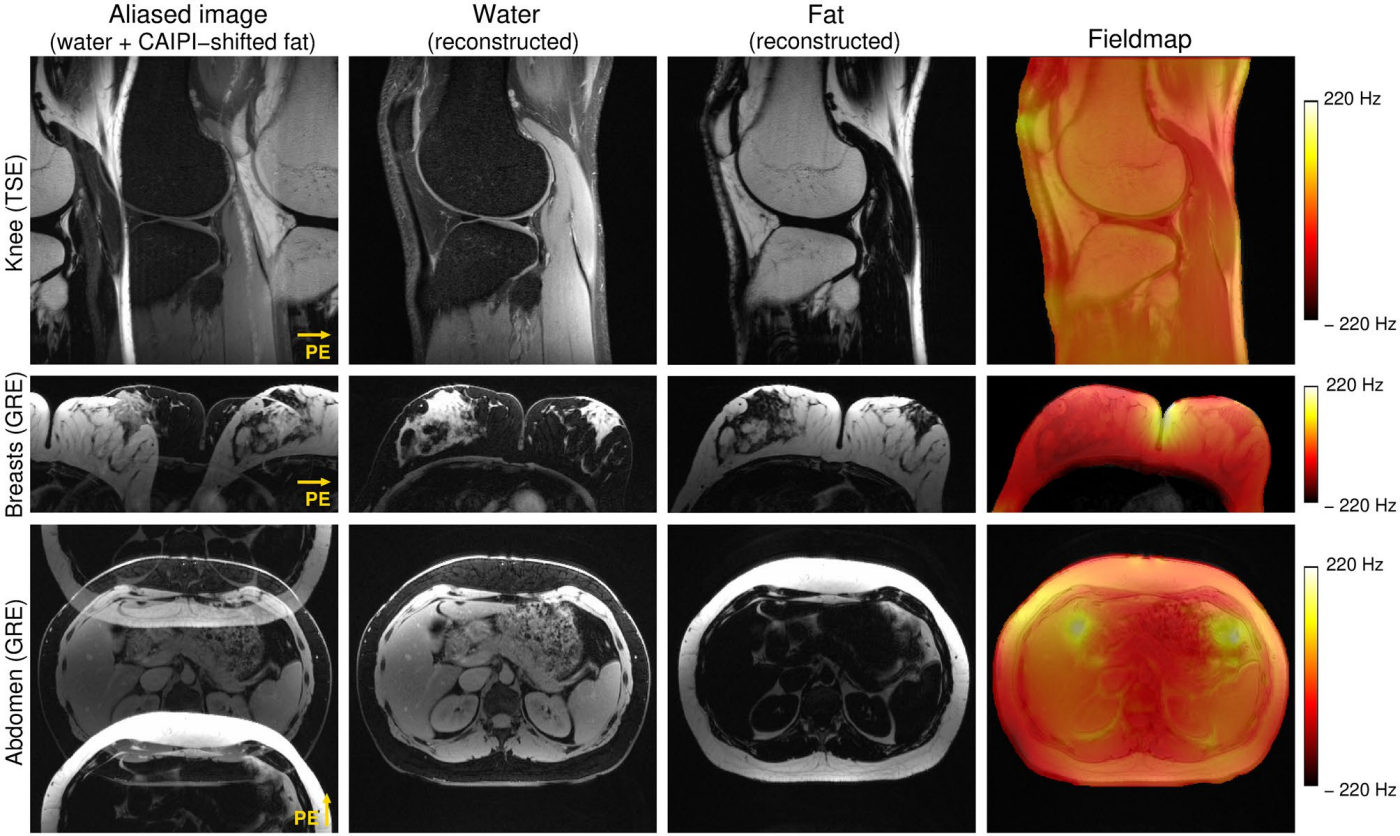
Excitation selectivity and unaliasing quality of Simultaneous Multiple Resonance Frequency imaging (SMURF) demonstrated for one exemplary volunteer for each body region under consideration. The acquired aliased images show the overlapping water and CAIPIRINHA‐shifted (along the phase‐encoding (PE) direction) fat images. Separate SMURF water and fat images, reconstructed from the aliased images, illustrate the achieved separation quality. (The separated images were rescaled for improved visibility.)

The performance of SMURF in all body regions examined is illustrated in Figure 5 for exemplary volunteers. For all body regions under consideration and all volunteers, the magnitude of local field deviations was below 220 Hz throughout the FOV and cleanly separated fat and water images were generated using SMURF. Artifacts from the unaliasing with slice‐GRAPPA were at the level of noise, evidenced by the near absence of water signal in the fatty areas (eg, bone marrow, adipose tissue) and vice versa (eg, muscles, breasts lobules, kidneys).

**FIGURE 6:**
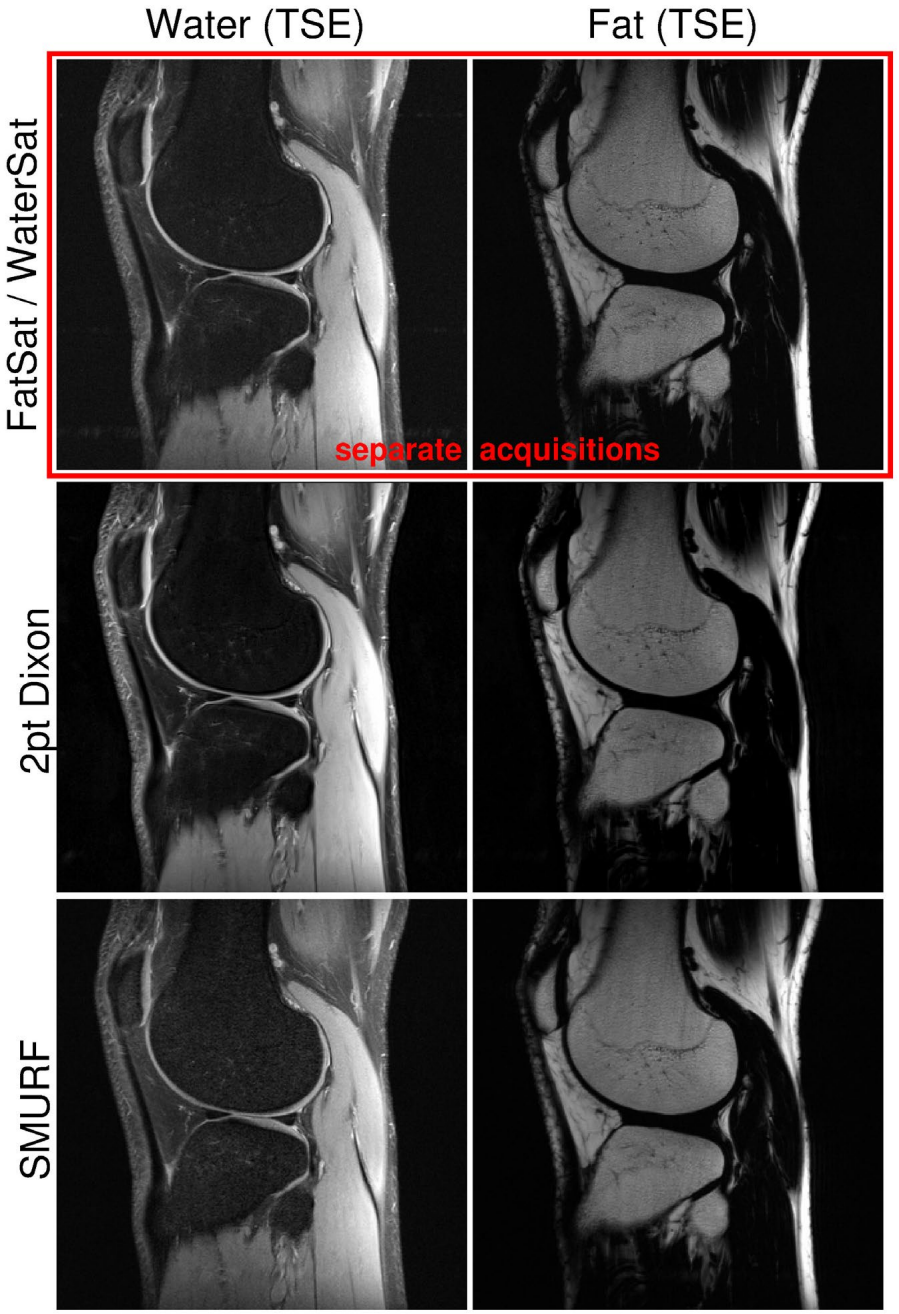
Comparison of knee 2D turbo spin echo water and fat images obtained using fat‐saturation and water‐saturation (top row), Dixon (middle row), and SMURF methods (bottom row), demonstrated for one exemplary volunteer. There is a high level of consistency between the methods. The SMURF water images show slightly higher signal in fatty‐tissue areas (eg, bones, subcutaneous fat). (The same non‐linear gray scales were used for all fat and all water images.)

**FIGURE 7:**
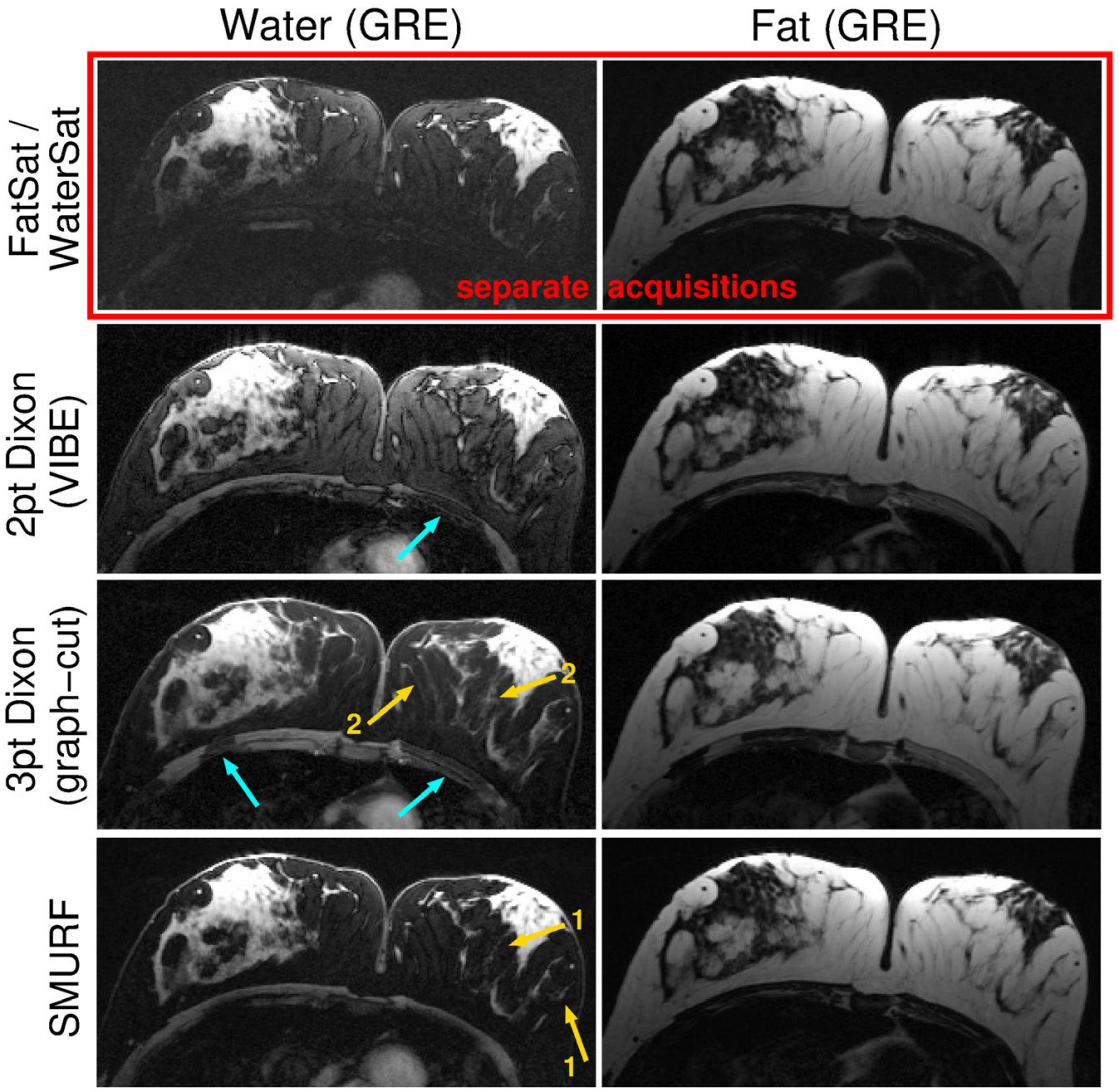
Comparison of GRE water and fat images of the breasts obtained using fat‐saturation and water‐saturation (top row), two‐point Dixon (second row), three‐point Dixon (third row), and SMURF methods (bottom row), demonstrated for one exemplary volunteer. Fat‐saturated images show some residual signal, low image SNR, and strong shading artifacts. Two‐point Dixon water images show very high residual fat signal obscuring the visibility of small water regions (eg, breast lobules, veins) surrounded by fatty tissues, that can be seen in the SMURF water images (gold arrows “1”). Three‐point Dixon images show even better separation quality, allowing a clear depiction of very small water structures (gold arrows “2”). In Dixon images, however, there is misattribution of water signal to the fat image, that is, fat‐water swaps (blue arrows), particularly in the three‐point Dixon images. The fat‐water attribution in SMURF images is, for all of these areas, correct. (Note that FatSat/WaterSat, three‐point Dixon and SMURF images were acquired using 2D imaging, while two‐point Dixon images were acquired using slab‐selective 3D imaging approach. The same non‐linear gray scales were used for all fat images and all water images.)

**FIGURE 8:**
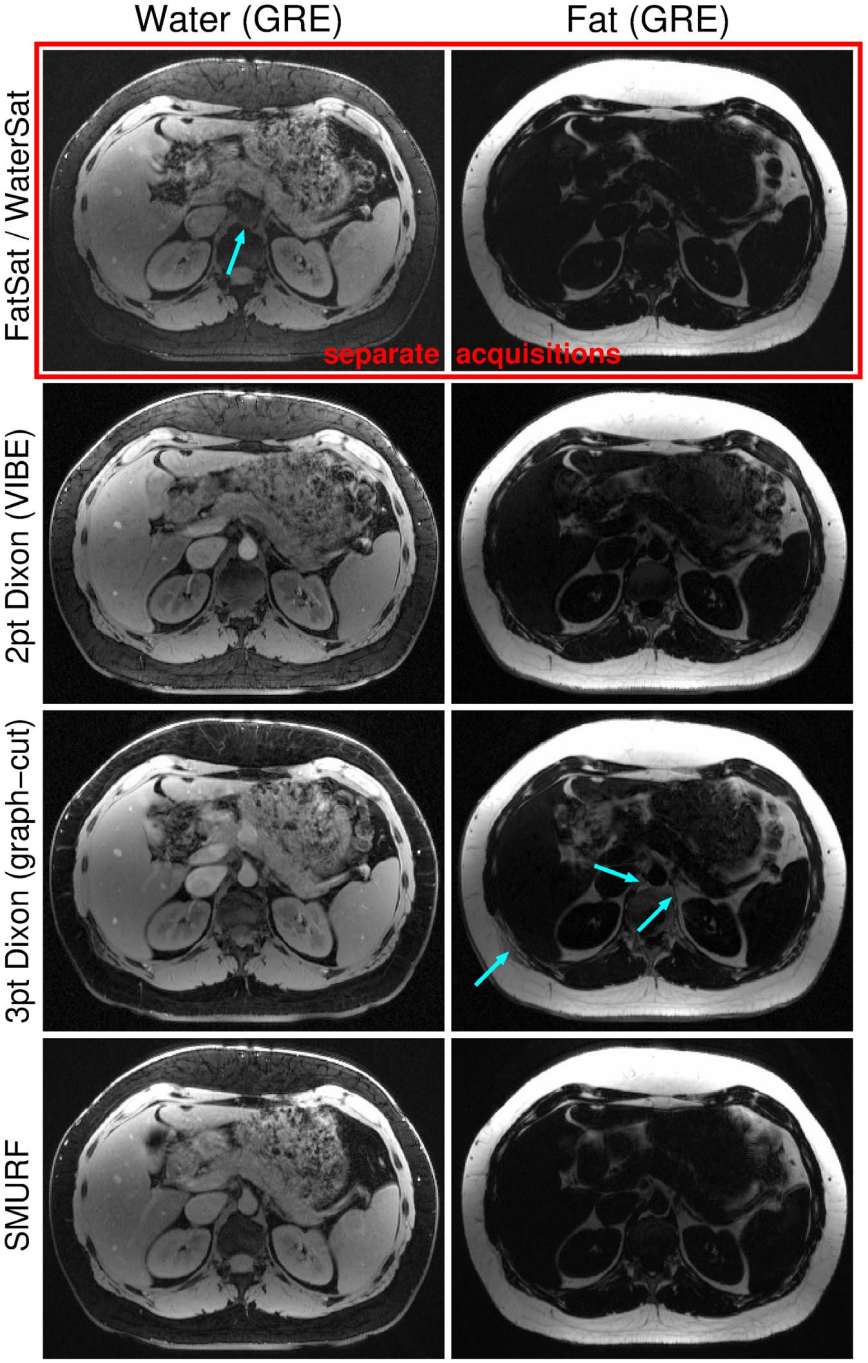
Comparison of abdominal GRE water and fat images obtained using fat‐saturation and water‐saturation (top row), two‐point Dixon (second row), three‐point Dixon (third row), and SMURF methods (bottom row), demonstrated for one exemplary volunteer. Fat‐saturated images show quite high residual signal and lower image SNR. Two‐point Dixon water images show very high residual fat signal, however, no fat‐water swaps. Three‐point Dixon images show clear fat‐water separation (very little residual signal), but several fat‐water swaps (blue arrows). SMURF images show very little residual signal and no fat‐water swaps. (Note that FatSat/WaterSat, three‐point Dixon and SMURF images were acquired using 2D imaging, while two‐point Dixon images were acquired using slab‐selective 3D imaging approach. The same non‐linear gray scale was used for all fat images and water images, respectively.)

Fat‐water separation achieved with SMURF is compared with water‐ and fat‐saturated acquisitions and with Dixon results for the knee, breasts, and abdomen in Figures 6, 7, 8 and Supporting Information Figures S3‐S6. The knee TSE SMURF images of fat and water were similar to the Dixon images and to the separately acquired water‐saturated and fat‐saturated images. With SMURF, there was a slight increase in signal attributed to water in fatty tissues, but the acquisition time was half that with the other methods (Figure 6, Supporting Information Figure S3).

In the breast GRE imaging (Figure 7, Supporting Information Figure S4), SMURF and fat‐saturation achieved better fat‐suppression than the two‐point Dixon, improving the visibility of small water regions (eg, breast lobules, veins) surrounded by fatty tissues. Fat‐saturated images showed low image SNR and strong shading artifacts, however. The three‐point Dixon achieved even better fat‐suppression, allowing the best depiction of very small water structures, but was more prone to fat‐water swaps (a misattribution of fat signal to the water image and vice versa).

In the abdominal GRE imaging (Figure 8, Supporting Information Figure S5), fat‐saturated images showed quite high residual signal and lower SNR. Two‐point Dixon water images showed high residual fat signal but no fat‐water swaps. Three‐point Dixon achieved clean fat‐water separation (very little residual signal), but some fat‐water swaps occurred for all volunteers. With SMURF, the fat‐water separation was quite clean and fat‐water attribution was correct (ie, it corresponded to the known tissue composition) in all subjects.

The performance of the two‐point and three‐point GRE Dixons and GRE SMURF was similar in the knee (Supporting Information Figure S6).

**FIGURE 9:**
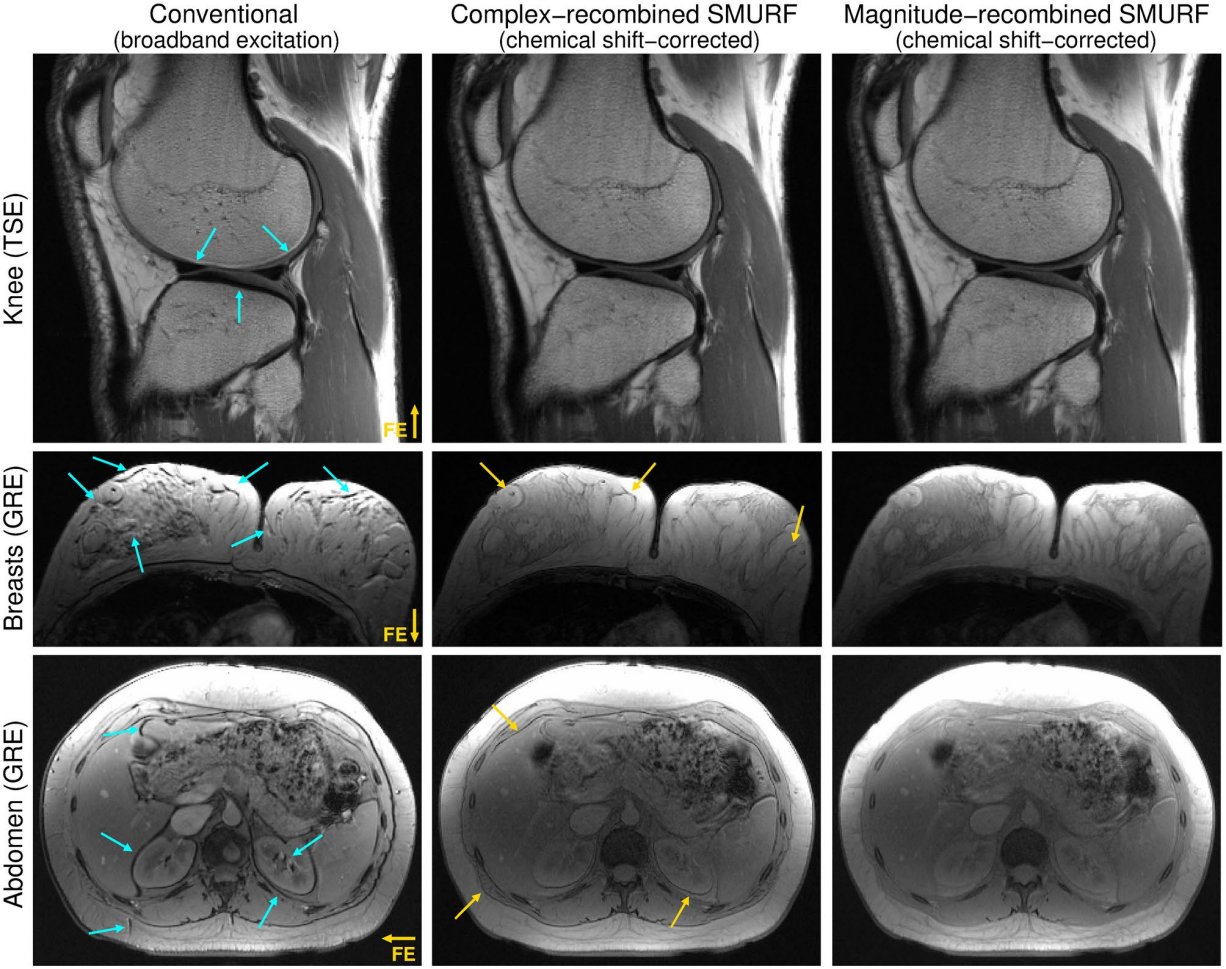
Comparison of conventional images (left) and chemical shift displacement‐corrected complex‐recombined (middle) and magnitude‐recombined (right) SMURF fat‐water images, illustrated for one exemplary volunteer for each body region under consideration. The SNR of the SMURF images is lower than that of the conventional images (expected reduction by a factor of $\sqrt{2}$ for complex‐recombined and by a factor of 2 for magnitude‐recombined images; see the Theory section), but the fat‐water misalignment is fully eliminated. Phase dispersion‐related signal cancellation is also removed in magnitude‐recombined GRE images (blue arrows). Note the slightly altered fat‐water contrast in SMURF GRE images resulting from the use of separate Ernst angles for fat and water.

The chemical shift displacement artifact was fully eliminated in recombined SMURF fat‐water images of a knee (TSE), breasts (GRE), and abdomen (GRE) (Figure 9). In GRE images, phase dispersion‐related signal cancellation was partially corrected in complex‐recombined SMURF images and fully corrected in magnitude‐recombined SMURF images. The SNR of recombined SMURF images was, however, decreased compared to conventional (broadband excitation) images.

## 5 DISCUSSION

A new fat‐water imaging method has been presented in gradient echo and turbo spin echo variants. The approach, Simultaneous Multiple Resonance Frequency imaging (SMURF), was shown to yield well‐separated fat and water images with minimal unaliasing artifacts or cross‐excitation, similar to those obtained with Dixon methods or using separate acquisitions with water‐saturation and fat‐saturation. SMURF, however, requires only one, single‐echo acquisition with echo times and receiver bandwidths, which can be chosen independently of chemical shift considerations and uses well‐established robust reconstruction methods. In common with the Dixon approach, combined fat‐water images free of chemical shift artifacts of both Type 1 (displacement) and Type 2 (complex signal cancellation) could also be generated.^17^, ^40^ In GRE imaging, any residual phase dispersion‐related fat‐water signal cancellation could be removed using magnitude recombination. Chemical shift corrections facilitate the assessment of the size and the relationship between fat‐based and water‐based structures and remove the requirement to choose the phase‐encoding direction so that chemical shift displacement causes minimum disturbance to structures of interest. In clinical imaging, for example of the joints, it is common to use fat‐saturation in many acquisitions but to acquire at least one set of images without fat‐sat to reveal image features in the fat signal. SMURF obviates the need for this additional scan, reducing the total measurement time.

SMURF can be combined with both partial Fourier and parallel imaging acceleration to reduce the acquisition time. As in SMS imaging, the maximum acceptable acceleration factor will generally be reduced compared to that which can be used in conventional (broadband excitation) imaging, as SMURF takes advantage of the spatially varying coil sensitives to separate fat and water. SMURF does not pose any special requirements on the reference data needed for fat‐water separation; the reference data commonly acquired in accelerated imaging (ie, a set of fully sampled central k‐space lines, “auto‐calibration scan”^27^) could be used.

The SMURF approach has an SNR benefit over broadband imaging in sequences that use non‐90° FAs (such as FLASH); the respective Ernst angles for the separately excited species can be used. Also, unlike in Dixon imaging, low receiver bandwidths can be used, resulting in a higher image SNR. On the other hand, parallel imaging reconstruction, applied to unalias fat and water, causes a (generally modest) SNR decrease by the g‐factor, as observed in SMS imaging.^24^ Recombination of fat and water images leads to an additional SNR decrease by a factor of $\sqrt{2}$, as explained in the Theory section. (The same SNR reduction affects recombined Dixon images.) In this study, this resulted in decreases in SNR in phantom images with SMURF reconstruction steps and also in a reduced SNR in the recombined SMURF TSE knee and GRE breast and abdominal images compared to the conventional images (broadband excitation with no spectral saturation).

Many sophisticated postprocessing methods have been developed for the Dixon approach.^8^ These differ greatly in the reliability of separation achieved and the requirements on the number and spacing of echoes. In this study, two approaches were applied for gradient echo imaging, and one for turbo spin echo imaging. The online reconstruction of the two‐point GRE Dixon data generated images with no fat‐water swaps, but with a high residual signal. On the other hand, the state‐of‐the‐art graph‐cut reconstruction^46^ of the three‐point GRE Dixon data achieved clean fat‐water separation (very little residual signal), but some fat‐water swaps were present in all measurements. The performance of the Dixon approaches may have been compromised, to some extent, by the comparatively long echo spacing (because of the monopolar readout). The online reconstruction of the two‐point TSE Dixon data resulted in a clean fat‐water separation and no fat‐water swaps, but the acquisition time was twice that of SMURF. As we postulated, the SMURF approach requires the B_0_ inhomogeneity to be less than half of the chemical shift difference between the two species, that is, 220 Hz for fat‐water imaging at 3 T, to achieve reliable separation. This was satisfied in most structures of interest, that is, the knee, breasts, and abdomen, allowing correct fat‐water separation using SMURF. It shows that although the Dixon approach should theoretically be less sensitive to field inhomogeneities than SMURF, it might not be the case in practice. In SMURF, the fat‐water swaps only occur where the shim is poor. In Dixon, even a single voxel with erroneous phase, caused by regional field inhomogeneity or flow effects, for instance, may lead to fat‐water swaps spanning large areas because of the use region‐growing and phase unwrapping methods. SMURF could be made yet more robust to field offsets by varying the frequencies of the spatial‐spectral pulses in line with variation in B_0_.^53^

A single RF band was used in SMURF excitation. The spectral complexity of fat resulted in a slightly reduced fat‐water suppression quality in SMURF compared to three‐point GRE and two‐point TSE Dixon results. Compared to the two‐point GRE Dixon approach, which is sensitive to B_0_ inhomogeneity and assumes no $T_{2}^{*}$ decay, and also compared to the separate acquisitions with water‐saturation and fat‐saturation, SMURF yielded improved separation, however.

To achieve high spectral selectivity and some insensitivity to field inhomogeneity, relatively long (11.76 ms) Shinnar‐Le Roux^33^, ^34^ excitation pulses were used in this study. The asymmetric, minimum phase design^35^ was used to reduce the echo time contribution of the RF pulse and, hence, allow shorter minimum TEs and higher maximum feasible echo train lengths. Nevertheless, in GRE Dixon imaging, several echoes could have been acquired over the same time and averaged to a final image. This led, in the in vivo GRE data acquired for the SNR comparison between SMURF and Dixon, to a theoretical SNR advantage by a factor of 1.7, which compensated for the 1.5× lower SNR in Dixon images due to the higher receiver bandwidths. The shorter TEs and, hence, less pronounced $T_{2}^{*}$ decay with Dixon led, however, together with the g‐factor‐related SNR losses and some reduction in fat signal due to the single‐peak assumption in SMURF to the observed higher SNR efficiency of Dixon compared to SMURF (by a factor of about 1.4 and 1.6 for water and fat tissues, respectively). To increase the highest achievable image SNR and SNR efficiency of SMURF, shorter RF pulses could be designed, either using more advanced pulse design approaches, such as optimal control^54^ and convex optimization^55^ or at the price of decreased spectral selectivity. In TSE imaging, the minimum echo spacing of SMURF (11.94 ms) was similar to that of conventional (broadband) acquisitions (11 and 12 ms with “fast” and “normal” RF, respectively) and shorter than with the two‐point Dixon (14 ms). The TR and TA of SMURF were also shorter than of Dixon; the TA of SMURF was half of Dixon's at the same TR, resulting in a decreased sensitivity to motion and flow artifacts and the possibility to use higher echo train lengths.

We have shown that the Simultaneous Multiple Resonance Frequency imaging approach can be used to concurrently generate separate images of fat and water and to correct chemical shift effects. In addition to facilitating the assessment of overlapping fat‐based and water‐based structures, it is expected to bring benefits for methods based on the signal phase, such as Quantitative Susceptibility Mapping, in which chemical shift effects lead to errors in field estimation.^56^ SMURF could also be used to quantify fat content^57^ and to calculate separate relaxation constants of fat and water in mixed tissues.^58^, ^59^, ^60^ The SMURF approach could also be used to simultaneously image other chemical species, such as PCr and PCi^61^ or hyperpolarized ^13^C pyruvate, lactate and alanine^62^, ^63^, ^64^ and could be extended to allow simultaneous imaging of three or more species.

## 6 CONCLUSION

We have presented a method that allows simultaneous, separate imaging of fat and water and the elimination of chemical shift artifacts in fat‐water images. The proposed Simultaneous Multiple Resonance Frequency (SMURF) method was implemented in gradient echo and turbo spin echo sequences and was shown to yield well‐separated fat and water images with minimal unaliasing artifacts or cross‐excitation in the knee, breasts and abdomen.

## Supporting information


**FIGURE S1** Dual‑band spatial‑spectral pulse used for excitation in 2D GRE‑SMURF imaging. A, RF waveform created by a train of sinc subpulses modulated by a dual‑band envelope. B, Waveform of the oscillating trapezoidal z‑gradient played concurrently with the RF pulse for slice‑selection. Excitation profile of the 30° pulse, comprising two frequency bands offset by 440 Hz, shown as a function of the position along the slice‐selection direction (C), the Larmor frequency (D), and the Larmor frequency and position along the slice‐selection direction (E)
**FIGURE S2 (with Table S1)** Comparison of SNR and SNR efficiency between 2D GRE a) SMURF, b) three‐point Dixon with long TR (same as SMURF) c) three‐point Dixon with short TR, assessed on the separated water and fat images. The shown SNR and SNR efficiency values represent the median of the values in all ROIs localized in the same areas and over the four repetitions. The SNR and SNR efficiency of SMURF is decreased compared to the Dixon approach – note the highest relative decrease in the liver
**FIGURE S3** Comparison of 2D turbo spin echo water and fat images of the knee obtained using fat‐saturation and water‐saturation (top row), Dixon (middle row), and SMURF methods (bottom row) for the two volunteers not shown in the main manuscript (Figure 6). There is a high level of consistency between the methods. SMURF water images show slightly higher signal in fatty‐tissue areas (eg, bones, subcutaneous fat), however, the acquisition time with SMURF was half of that with Dixon and with separate acquisitions with fat‐saturation and water‐saturation respectively. (The same non‐linear grey scales were used for all fat and all water images.)
**FIGURE S4** Comparison of gradient echo water and fat images of the breasts obtained using fat‐saturation and water‐saturation (top row), two‐point Dixon (second row), three‐point Dixon (third row) and SMURF (bottom row) for the two volunteers not shown in the main manuscript (Figure 7). Fat‐saturated images show some residual signal, low image SNR and strong shading artefacts. Two‐point Dixon water images show very high residual fat signal, obscuring the visibility of small water regions (eg, breast lobules, veins) surrounded by fatty tissues, that can be seen in the SMURF water images (gold arrows “1”). Three‐point Dixon images show even better separation quality, allowing the best depiction of very small water structures (gold arrows “2”). In Dixon images, mainly in the three‐point images, there is a misattribution of water signal to the fat image, that is, fat‐water swaps (blue arrows). The fat‐water assignment in SMURF images is correct for all of these areas. (Note that FatSat/WaterSat, three‐point Dixon and SMURF images were acquired using 2D imaging, while two‐point Dixon images were acquired using slab‐selective 3D imaging approach. The same non‐linear grey scales were used for all fat images and all water images.)
**FIGURE S5** Comparison gradient echo water and fat images of the abdomen obtained using fat‐saturation and water‐saturation (top row), two‐point Dixon (second row), three‐point Dixon (third row) and SMURF (bottom row) for the two volunteers not shown in the main manuscript (Figure 8). Fat‐saturated images show quite high residual signal and lower image SNR. Two‐point Dixon water images show high residual fat signal and artefacts at the edges of some tissue boundaries (gold arrows), but no fat‐water swaps. Three‐point Dixon images show clear fat‐water separation (very little residual signal), but several fat‐water swaps (blue arrows). SMURF images show very little residual signal and no fat‐water swaps. (Note that FatSat/WaterSat, three‐point Dixon and SMURF images were acquired using 2D imaging, while two‐point Dixon images were acquired using slab‐selective 3D imaging approach. The same non‐linear grey scales were used for all fat images and all water images.)
**FIGURE S6** Comparison of gradient echo water and fat knee images obtained using two‐point Dixon (top row), three‐point Dixon (middle row) and SMURF methods (bottom row), shown both in 3D (left column) and 2D (right column) imaging. Two‐point Dixon water images show high residual fat signal, but no fat‐water swaps. Three‐point Dixon images show clear fat‐water separation (very little residual signal), but several fat‐water swaps (blue arrows). SMURF images show clear fat‐water separation and no fat‐water swaps. (The same non‐linear grey scales were used for all fat images and all water images.)

## Data Availability

The data that support the findings of this study are openly available in “SMURF (raw MRI data)” and “SMURF (MRI images)” at https://doi.org/10.7910/DVN/XNMCYI and https://doi.org/10.7910/DVN/TMPDCR, references [65] and [66], respectively.
