# Supplementary material for: Simultaneous Multiple Resonance Frequency imaging (SMURF): Fat‐water imaging using multi‐band principles
Source: Magn Reson Med. 2020 Sep 27;85(3):1379–96. doi: 10.1002/mrm.28519 (PMC7756227; doi:10.1002/mrm.28519)
Supplement: Supplementary file 1 — FIGURE S1 Dual‑band spatial‑spectral pulse used for excitation in 2D GRE‑SMURF imaging. A, RF waveform created by a train of sinc subpulses modulated by a dual‑band envelope. B, Waveform of the oscillating trapezoidal z‑gradient played concurrently with the RF pulse for slice‑selection. Excitation profile of the 30° pulse, comprising two frequency bands offset by 440 Hz, shown as a function of the position along the slice‐selection direction (C), the Larmor frequency (D), and the Larmor frequency and position along the slice‐selection direction (E) FIGURE S2 (with Table S1) Comparison of SNR and SNR efficiency between 2D GRE a) SMURF, b) three‐point Dixon with long TR (same as SMURF) c) three‐point Dixon with short TR, assessed on the separated water and fat images. The shown SNR and SNR efficiency values represent the median of the values in all ROIs localized in the same areas and over the four repetitions. The SNR and SNR efficiency of SMURF is decreased compared to the Dixon approach – note the highest relative decrease in the liver FIGURE S3 Comparison of 2D turbo spin echo water and fat images of the knee obtained using fat‐saturation and water‐saturation (top row), Dixon (middle row), and SMURF methods (bottom row) for the two volunteers not shown in the main manuscript (Figure 6). There is a high level of consistency between the methods. SMURF water images show slightly higher signal in fatty‐tissue areas (eg, bones, subcutaneous fat), however, the acquisition time with SMURF was half of that with Dixon and with separate acquisitions with fat‐saturation and water‐saturation respectively. (The same non‐linear grey scales were used for all fat and all water images.) FIGURE S4 Comparison of gradient echo water and fat images of the breasts obtained using fat‐saturation and water‐saturation (top row), two‐point Dixon (second row), three‐point Dixon (third row) and SMURF (bottom row) for the two volunteers not shown in the main manuscript (Figure 7). Fat‐saturate [file MRM-85-1379-s001.docx]

***Supporting Information Figure S1***

Two least‑squares filtered minimum‑phase Shinnar‑Le Roux pulses^1–3^ of duration 11.76 ms were designed and used to create both bands of the dual‑band pulses employed for *in vivo* measurements performed in this study. One, optimized for excitation at 90°, was used in TSE imaging and is illustrated in Figure 1. The second, designed for excitation at 30°, was used in GRE imaging and is shown in Supporting Information Figure S1. The echo time contributions (i.e. isodelay), as well as passbands and transition bands of these, differed: 1.4 ms and circa 145 Hz and 90 Hz for the RF pulse optimized for a 90° excitation in TSE imaging, and 3.64 ms and circa 115 Hz and 120 Hz for the RF pulse optimized for a 30° excitation in GRE imaging respectively.


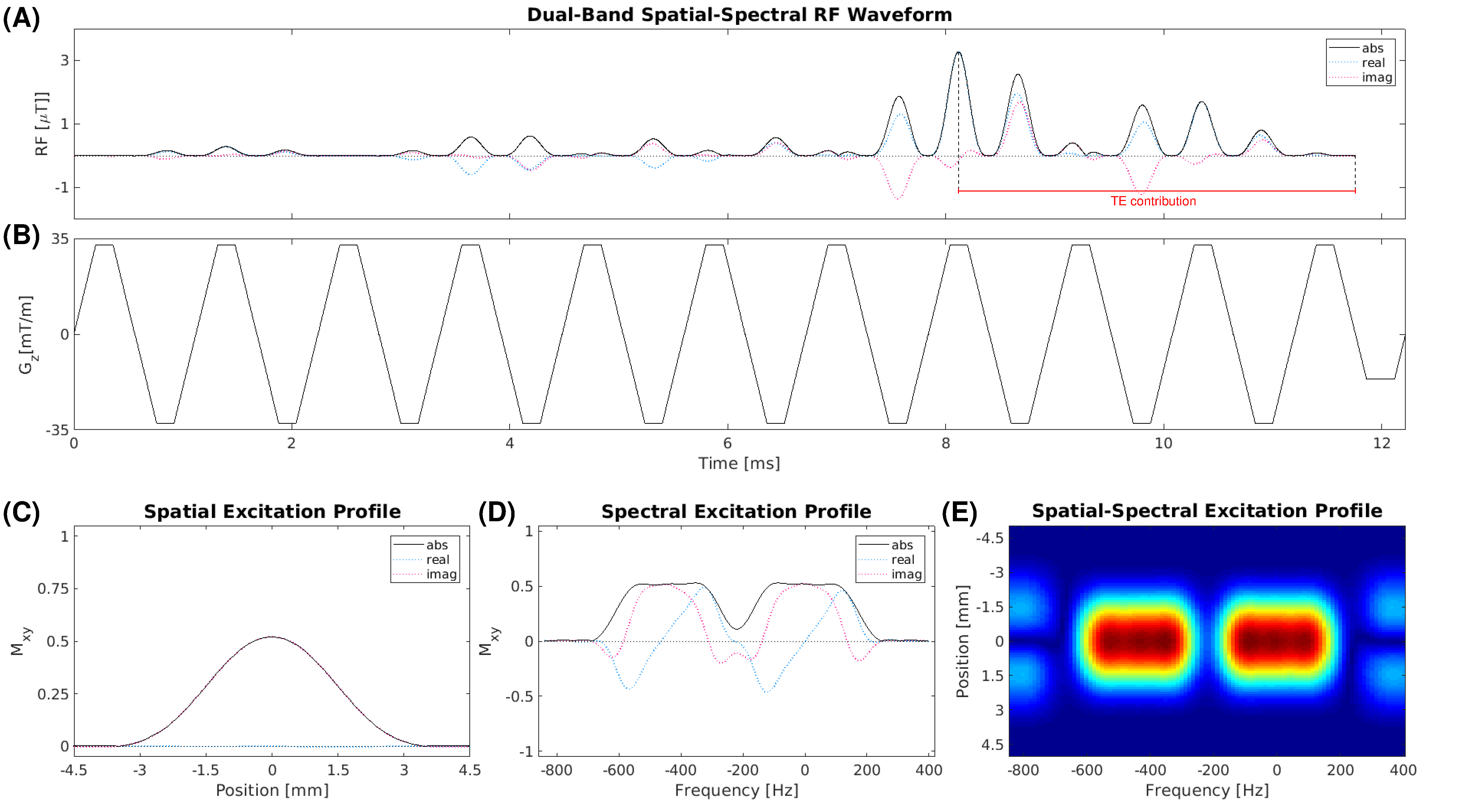


*Supporting Information Figure S1: Dual‑band spatial‑spectral pulse used for excitation in 2D GRE‑SMURF imaging. (A) RF waveform created by a train of sinc subpulses modulated by a dual‑band envelope. (B) Waveform of the oscillating trapezoidal z‑gradient played concurrently with the RF pulse for slice‑selection. Excitation profile of the 30° pulse, comprising two frequency bands offset by 440 Hz, shown as a function of the position along the slice‐selection direction (C), the Larmor frequency (D), and the Larmor frequency and position along the slice‐selection direction (E).*

***Supporting Information Figure S2***

There are several differences between SMURF and Dixon sequences that lead to disparities in SNR and SNR efficiency (given as $SNR/\sqrt{scan time}$) between the two. In Dixon imaging, several echoes contribute to the final image, increasing the SNR of the final image by a factor of $\sqrt{N_{echo}}$. To increase the efficiency and reduce phase errors, this requires the use of short echo spacing and hence high receiver bandwidths, leading to a compensatory decrease in SNR. To a first approximation, though, image SNR is proportional to the square root of the readout duration, whether this is spent acquiring one or multiple echoes. The SMURF approach has a slight SNR benefit in sequences that use non‑90° flip angles (such as FLASH) in that the respective Ernst angles for the separately excited species can be used. On the other hand, because of the single fat‑peak assumption in SMURF (using a single RF pulse to excite fat with several spectral components actually contributing to its signal), some signal and hence SNR losses occur in fat. The parallel imaging reconstruction applied in SMURF to unalias fat and water also causes some, typically modest, SNR decrease, given by the g‑factor. Additionally, the generally longer duration of spectrally‑selective RF pulses limits the minimum TE and, because of T_2_ or T_2_^*^ decay, the maximum achievable SNR in SMURF. The asymmetric, 11.76 ms minimum phase SLR pulse was used in this study to decrease the minimum echo times while reaching high spectral selectivity (the echo time contribution of the 11.76 ms minimum phase spatial-spectral SLR pulse used for gradient echo imaging was 3.64 ms).

To compare the SNR and SNR efficiency of SMURF with the Dixon method *in vivo* (abdomen), the difference method^4^ was used. The SNR was calculated over two identical scans measured in a succession (“a pair”). The SNR and SNR efficiency of 2D GRE SMURF images of TE = 6.73 ms and TR = 110 ms was compared with two sets of 3‑point 2D GRE Dixon images; (a) “long‑TR Dixon” – the same TR (110 ms) as for SMURF images and (b) “short‑TR Dixon” – the minimum possible TR (67 ms), all with respective flip angles and TE = {2.2, 5.3, 8.4} ms. Although the SNR of the long‑TR Dixon is expected to be higher than that of the short‑TR Dixon because of the higher flip angle, their SNR efficiencies should be similar.^5^ The short‑TR Dixon should, however, be less affected by motion and flow artefacts, which might potentially lead to an increase in SNR efficiency.

Since the difference method is sensitive to motion between the pairs of scans, four pairs of scans were measured in pseudo-random order for each method, and median SNR values were calculated. The SNR was assessed on a number of slices of the separated water and fat images over 40 manually defined ROIs of the same size (100 voxels), defined in areas in which there were no fat‑water swaps and which were least affected by motion. Twenty ROIs were defined on the water images in water‑dominant tissue areas: (a) ROIs 1‑5 in the right erector spinae muscle, (b) ROIs 6‑10 in the left erector spinae muscle, (c) ROIs 11‑15 in the liver and (d) ROIs 16‑20 in the left kidney and the other twenty ROIs were defined on the fat images in the fat‑dominant tissue areas: (a) ROIs 21‑25 in subcutaneous fat on dorsal right body-side, (b) ROIs 26‑30 in subcutaneous fat on dorsal left body-side, (c) ROIs 31‑35 in visceral fat around the right kidney and (d) ROIs 36‑40 in visceral fat around the left kidney. Medians of the SNR and SNR efficiency values in all ROIs localized in the same areas and over the four repetitions were calculated and compared between the methods.

Lower SNR and SNR efficiency in ROIs 11‑20 and 31‑40 were observed for all methods due to decreased coil sensitivities and increased susceptibility to motion between the image pairs (the ROIs were further away from stable back areas). In all water ROIs except the liver, the SNR of SMURF was similar to that of short‑TR Dixon and slightly than that of the long‑TR Dixon – the median decreased by a factor of 1.1 (Supporting Information Table S1). In the liver (ROIs 11‑15), a more pronounced SNR decrease was observed, with the medians being decreased by factors of 1.4 and 1.6, respectively – due to generally faster T_2_^*^ decay in the liver than in other tissues (T_2_^*^ values were, however, not assessed). Similarly, the SNR efficiency of SMURF in the water ROIs was decreased compared to the short‑TR Dixon by a factor of 1.4 and compared to the long‑TR Dixon by a factor of 1.1. In the liver, the SNR efficiency was decreased by factors of 1.7 and 1.6, respectively. In the fat ROIs, a larger relative decrease in SNR and SNR efficiency between SMURF and Dixon methods was observed – median SNR being decreased by factors of 1.2 and 1.4 compared to the short‑TR and long‑TR Dixon respectively, corresponding to the decrease in SNR efficiencies by factors of 1.6 and 1.4.

| **SNR / SNR efficiency *in vivo* (abdomen)** | | | | | | | | | |
| --- | --- | --- | --- | --- | --- | --- | --- | --- | --- |
|  | **Water** | | | |  | **Fat** | | | |
|  | Erector spinae muscle  (ROIs 1-5) | Erector spinae muscle  (ROIs 6-10) | Liver  (ROIs 11-15) | Kidneys  (ROIs 16-20) |  | Subcut. fat  (ROIs 21-25) | Subcut. fat  (ROIs 26-30) | Visceral fat  (ROIs 31-35) | Visceral fat  (ROIs 36-40) |
| SMURF  (TA = 26.3 s) | 66.97 / 13.06 | 60.99 / 11.89 | 32.94 / 6.43 | 31.35 / 6.11 |  | 89.88 / 17.53 | 58.14 / 14.32 | 42.18 / 8.23 | 35.76 / 6.97 |
| Long-TR Dixon  (TA = 26.3 s) | 73.83 / 14.40 | 66.77 / 13.02 | 51.94 / 10.13 | 36.61 / 7.14 |  | 123.90 / 24.16 | 85.65 / 16.70 | 54.50 / 10.63 | 48.04 / 9.37 |
| Short-TR Dixon  (TA = 16.0 s) | 63.82 / 15.95 | 59.76 / 14.94 | 44.19 / 11.04 | 31.27 / 7.82 |  | 108.88 / 27.21 | 77.26 / 19.31 | 49.18 / 12.29 | 40.51 / 10.12 |

*Supporting Information Table S1: Comparison of SNR and SNR efficiency between 2D GRE (a) SMURF, (b) three‑point Dixon with long TR (same as SMURF) (c) three‑point Dixon with short TR, assessed on the separated water and fat images. The shown SNR and SNR efficiency values represent the median of the values in all ROIs localized in the same areas and over the four repetitions. The SNR and SNR efficiency of SMURF is decreased compared to the Dixon approach – note the highest relative decrease in the liver.*

***Supporting Information Figure S3
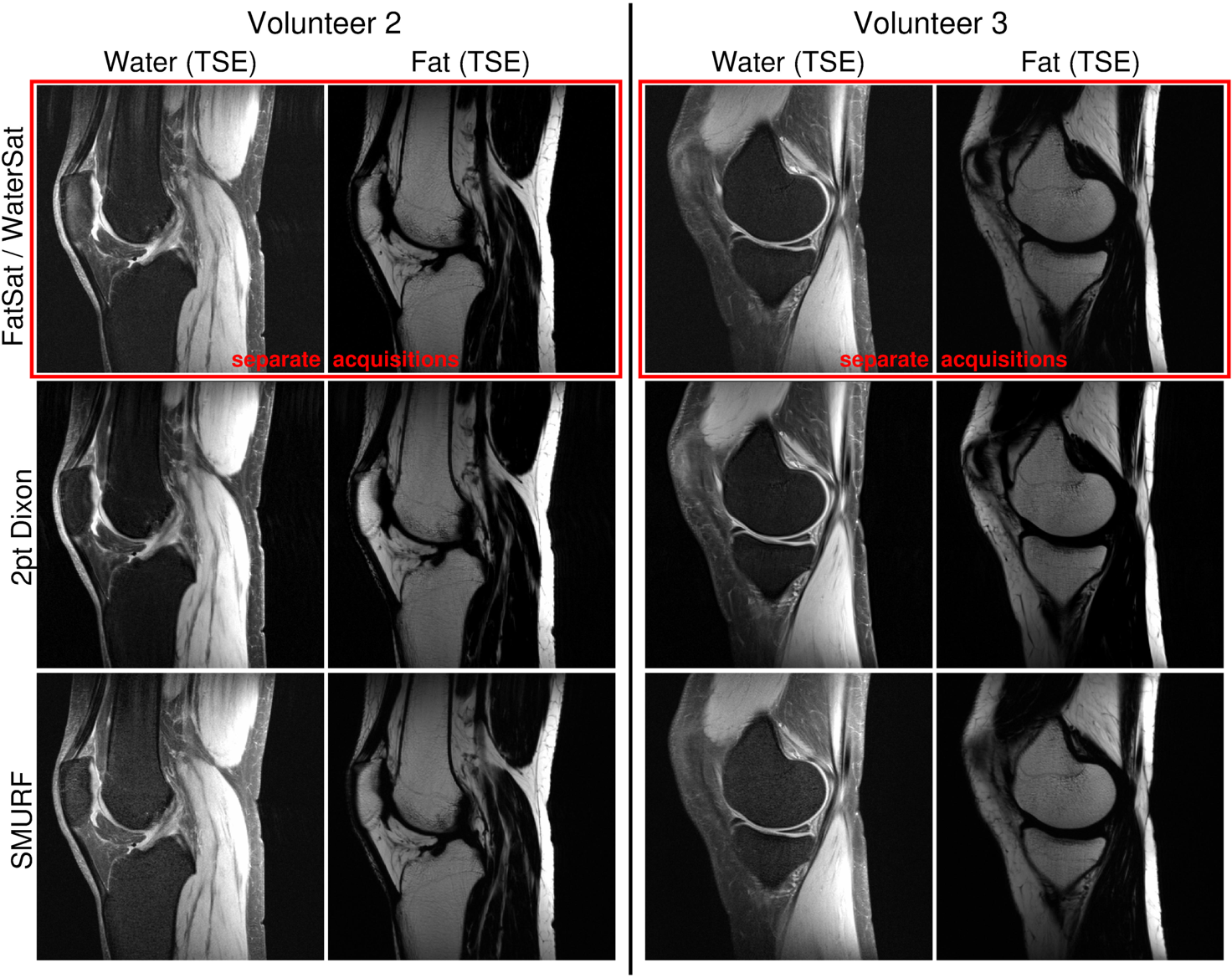
***

*Supporting Information Figure S3: Comparison of 2D turbo spin echo water and fat images of the knee obtained using fat‑saturation and water‑saturation (top row), Dixon (middle row), and SMURF methods (bottom row) for the two volunteers not shown in the main manuscript (Figure 6). There is a high level of consistency between the methods. SMURF water images show slightly higher signal in fatty-tissue areas (e.g. bones, subcutaneous fat), however, the acquisition time with SMURF was half of that with Dixon and with separate acquisitions with fat‑saturation and water‑saturation respectively. (The same non‑linear grey scales were used for all fat and all water images.)*

***Supporting Information Figure S4***

***
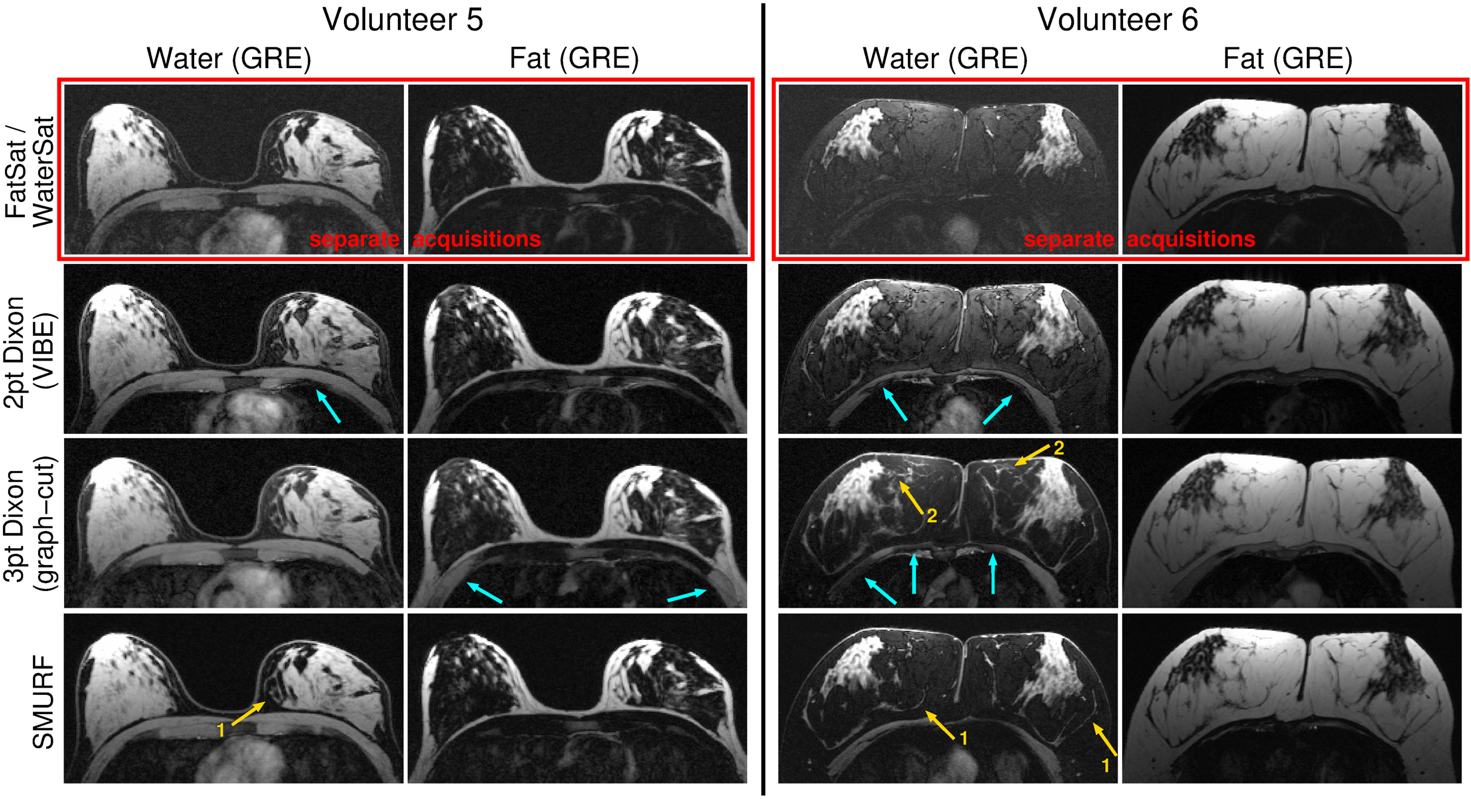
***

*Supporting Information Figure S4: Comparison of gradient echo water and fat images of the breasts obtained using fat‑saturation and water‑saturation (top row), two‑point Dixon (second row), three‑point Dixon (third row) and SMURF (bottom row) for the two volunteers not shown in the main manuscript (Figure 7). Fat‑saturated images show some residual signal, low image SNR and strong shading artefacts. Two‑point Dixon water images show very high residual fat signal, obscuring the visibility of small water regions (e.g. breast lobules, veins) surrounded by fatty tissues, that can be seen in the SMURF water images (gold arrows “1”). Three‑point Dixon images show even better separation quality, allowing the best depiction of very small water structures (gold arrows “2”). In Dixon images, mainly in the three‑point images, there is a misattribution of water signal to the fat image – fat‑water swaps (blue arrows). The fat‑water assignment in SMURF images is correct for all of these areas. (Note that FatSat/WaterSat, three‑point Dixon and SMURF images were acquired using 2D imaging, while two‑point Dixon images were acquired using slab‑selective 3D imaging approach. The same non‑linear grey scales were used for all fat images and all water images.)*

***Supporting Information Figure S5***

*
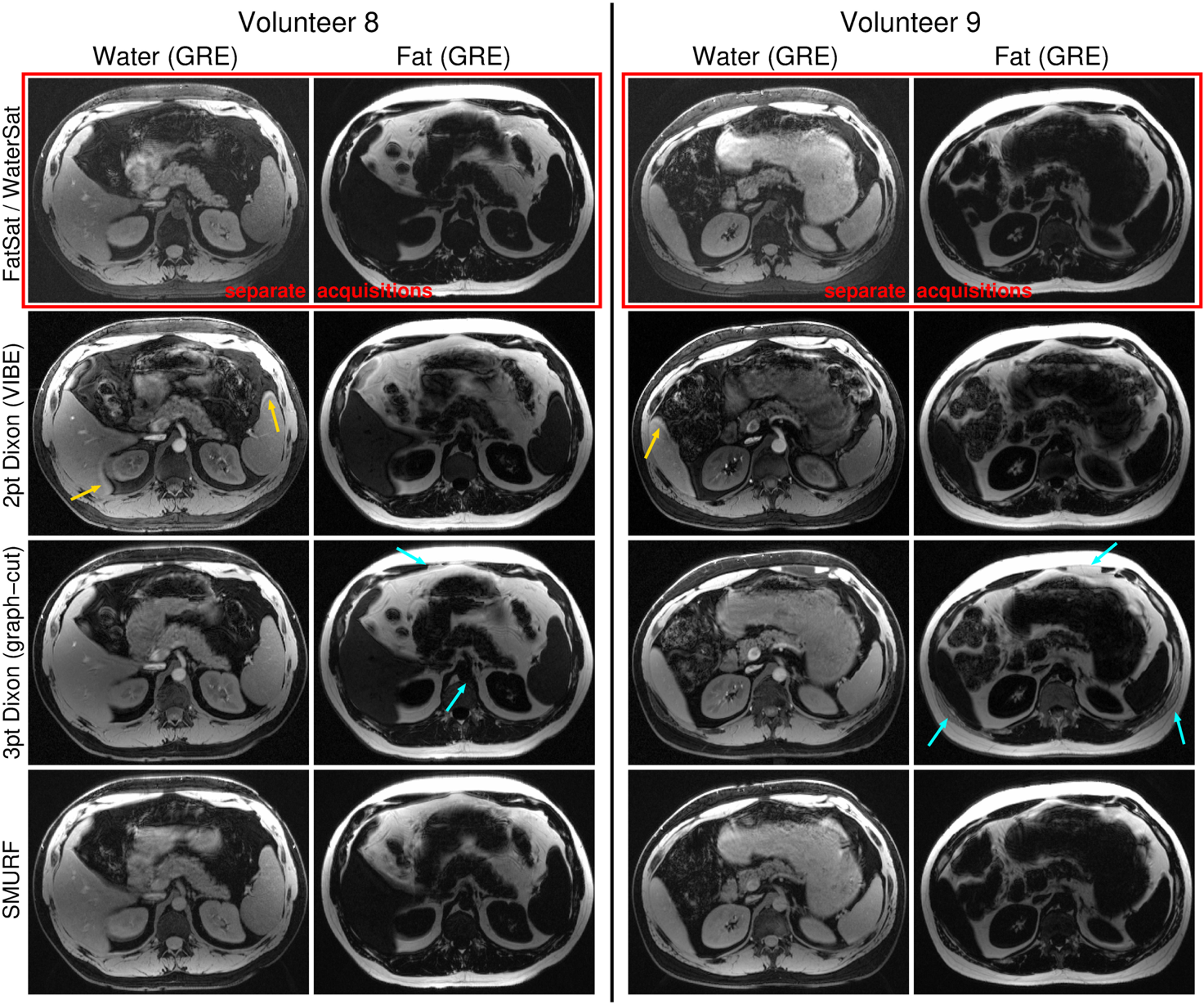
*

*Supporting Information Figure S5: Comparison gradient echo water and fat images of the abdomen obtained using fat‑saturation and water‑saturation (top row), two‑point Dixon (second row), three‑point Dixon (third row) and SMURF (bottom row) for the two volunteers not shown in the main manuscript (Figure 8). Fat‑saturated images show quite high residual signal and lower image SNR. Two‑point Dixon water images show high residual fat signal and artefacts at the edges of some tissue boundaries (gold arrows), but no fat‑water swaps. Three‑point Dixon images show clear fat‑water separation (very little residual signal), but several fat‑water swaps (blue arrows). SMURF images show very little residual signal and no fat‑water swaps. (Note that FatSat/WaterSat, three‑point Dixon and SMURF images were acquired using 2D imaging, while two‑point Dixon images were acquired using slab‑selective 3D imaging approach. The same non‑linear grey scales were used for all fat images and all water images.)*

***Supporting Information Figure S6***

The comparison between SMURF and Dixon is, in the main manuscript, shown for gradient echo (GRE) imaging of breasts and abdomen, and turbo spin echo (TSE) imaging of a knee. Although GRE imaging of a knee is seldom in clinical practice, here we show this additional comparison between SMURF and Dixon, to confirm the performance of the two methods. Unlike the breast and abdomen measurements, in which breathing restricted the maximum number of slices which could be acquired and motion of bowels and heart might have decreased the fat‑water separation quality, the knee data could be acquired with protocols which were well matched between methods, and the measurements were largely unaffected by motion.

As mentioned in the manuscript, the only available GRE sequence that allows online Dixon reconstruction on our scanner (*syngo* MR VE11C) is the 3D two‑point VIBE.^6^ Therefore, as for the GRE comparison in breasts and abdomen, here we have acquired: (a) 3D two‑point GRE Dixon images using the VIBE sequence, and (b) 2D three‑point images using standard (non-Dixon) Siemens product GRE sequence for offline reconstruction. Additionally, (c) 3D three‑point images for offline reconstruction were acquired. The offline Dixon reconstruction was performed using the graph‑cut approach^7^ from the Fat‑water Toolbox.^8^

Sagittal GRE images were measured with anterior-posterior phase‑encoding direction, FOV = 180 x 180 mm, in‑plane resolution = 0.75 x 0.75 mm, 44 slices, and using phase partial Fourier factor of 6/8. All (non‑selective) 3D images were acquired with a 3.3 mm slice thickness and all 2D images with a 3 mm slice thickness and 10% slice gap. The 3D SMURF images were acquired with TE = 6.68 ms, TR = 19 ms, TA = 2:40 min, rBW/pixel = 220 Hz, and using respective Ernst angles for fat and water bands, FA(fat) = 17° and FA(water) = 9°. The (a) 3D two‑point Dixon images were acquired with TE = {2.27, 5.67} ms, TR = 9 ms, TA = 1:03 min, rBW/pixel = 515 Hz, FA = 9° (mean of fat and water Ernst angles) and the (c) 3D three‑point Dixon images were acquired with TE = {2.2, 5.35, 8.5} ms, TR = 11 ms, TA = 1:32 min, rBW/pixel = 630 Hz and FA = 10° (mean of fat and water Ernst angles). The 2D SMURF images were acquired with TE = 7.02 ms, TR = 832 ms, TA = 2:38 min, rBW/pixel = 220 Hz, and using respective Ernst angles for fat and water bands, FA(fat) = 83° and FA(water) = 56°. The (b) 2D three‑point Dixon images were acquired with TE = {2.2, 5.3, 8.4} ms, TR = 500 ms, TA = 1:35 min, rBW/pixel = 630 Hz, and FA = 59° (mean of fat and water Ernst angles).

Similar as in breasts and abdomen, two‑point Dixon water images showed high residual fat signal, but no fat‑water swaps. Three‑point Dixon images, both 2D and 3D variants, showed clear fat‑water separation (very little residual signal), but several fat‑water swaps have occurred. SMURF images showed clear fat‑water separation and no fat‑water swaps. The Dixon images provided relatively higher signal in bones, due to the contribution of short‑TE acquisitions (first and second echoes were acquired at shorter TE than the TE of SMURF), however, the images were more blurred due to the averaging of three echoes into the final image of fat or water, respectively.

*
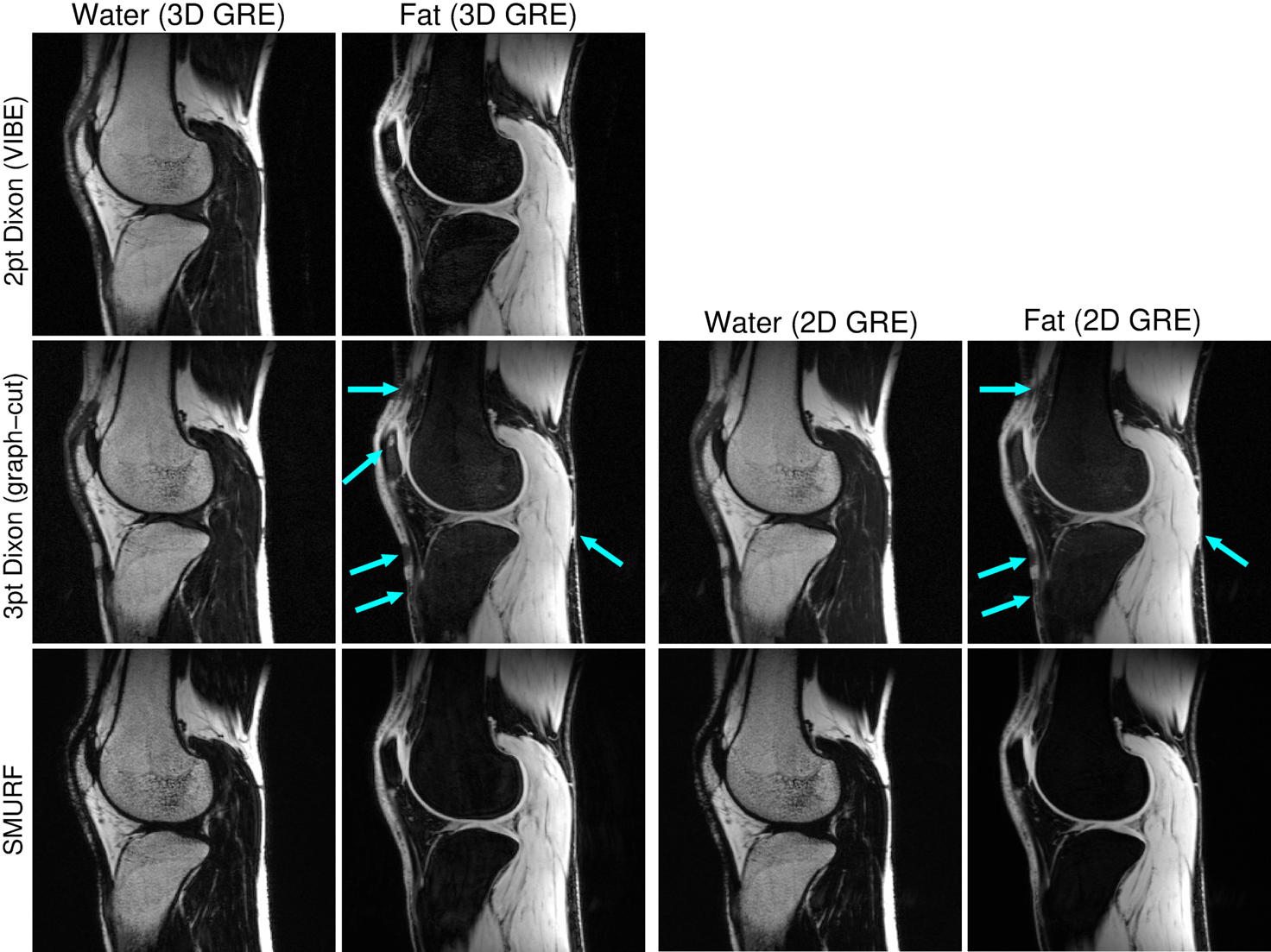
*

*Supporting Information Figure S6: Comparison of gradient echo water and fat knee images obtained using two‑point Dixon (top row), three‑point Dixon (middle row) and SMURF methods (bottom row), shown both in 3D (left column) and 2D (right column) imaging. Two‑point Dixon water images show high residual fat signal, but no fat‑water swaps. Three‑point Dixon images show clear fat‑water separation (very little residual signal), but several fat‑water swaps (blue arrows). SMURF images show clear fat‑water separation and no fat‑water swaps. (The same non‑linear grey scales were used for all fat images and all water images.)*

***References:***

1. Shinnar M, Eleff S, Subramanian H, Leigh JS. The synthesis of pulse sequences yielding arbitrary magnetization vectors. *Magn Reson Med*. 1989;12(1):74-80.

2. Le Roux P. Exact synthesis of radio frequency waveforms. In: *Proceedings of the 7th Annual Meeting of SMRM*, San Francisco, CA, USA, 1988, p. 1049

3. Pauly J, Le Roux P, Nishimura D, Macovski A. Parameter relations for the Shinnar-Le Roux selective excitation pulse design algorithm. *IEEE Trans Med Imaging*. 1991;10(1):53-65. doi:10.1109/42.75611

4. Goerner FL, Clarke GD. Measuring signal-to-noise ratio in partially parallel imaging MRI. *Med Phys*. 2011;38(9):5049-5057. doi:10.1118/1.3618730

5. Helms G, Dechent P. Increased SNR and reduced distortions by averaging multiple gradient echo signals in 3D FLASH imaging of the human brain at 3T. *J Magn Reson Imaging*. 2009;29(1):198-204. doi:10.1002/jmri.21629

6. Rofsky NM, Lee VS, Laub G, et al. Abdominal MR imaging with a volumetric interpolated breath-hold examination. *Radiology*. 1999;212(3):876-884. doi:10.1148/radiology.212.3.r99se34876

7. Hernando D, Kellman P, Haldar JP, Liang Z-P. Robust Water/Fat Separation in the Presence of Large Field Inhomogeneities Using a Graph Cut Algorithm. *Magn Reson Med*. 2010;63(1):79-90. doi:10.1002/mrm.22177

8. ISMRM Fat-water toolbox. ISMRM fat-water separation workshop 2012. “http://www.ismrm.org/workshops/FatWater12/.” Accessed July 29, 2020
